# Stakeholder-Reported Requirements for Future Artificial Intelligence Decision Support in Periodontal Care: A Cross-Sectional Survey of Dentists, Physicians and Patients in Kazakhstan

**DOI:** 10.3390/healthcare14203554

**Published:** 2026-10-21

**Authors:** Yerbol Ayash, Aigul Ismailova, Anar Aidarkhanova, Aiman Mussina, Zhanat Abdikadyr, Akerke Chayakova

**Affiliations:** Center of Epidemiology and Management, Astana Medical University, Astana010000 , Kazakhstan; 202514618@amu.kz (Y.A.); ismailova.a@amu.kz (A.I.); aidarkhanova.a@amu.kz (A.A.); mussina.a@amu.kz (A.M.); abdikadyr.zh@amu.kz (Z.A.)

**Keywords:** artificial intelligence, periodontal diseases, periodontal screening, clinical decision support systems, primary health care, survey research, stakeholder survey, Kazakhstan, human–AI collaboration, medical liability

## Abstract

**Highlights:**

**What are the main findings?**

• Dentists’ choices were spread across five autonomy options that all kept the clinician in control (no autonomous option was offered); low autonomy was the most frequent but a minority choice (30.3%), and the absence of a legal framework and liability insurance was the barrier most often cited (60.6%).
• Among adult patient respondents recruited through clinical and professional channels and an inter-university platform, about half reported at least one periodontal sign. Associations with high stress and a positive family history were exploratory, sensitive to data-quality and outcome-definition decisions, and lost significance after exclusion of a 204-response block whose eligibility could not be verified; periodontal eligibility was not clinically verified.

**What are the implications of the main findings?**

• The clinician surveys suggest preliminary requirements for a future periodontal AI tool, such as early gingivitis detection and interprofessional data exchange; a simple screening mode for primary care physicians is a hypothesis derived from their reported barriers and needs testing.
• Dentists’ most frequently cited barrier—uncertain legal liability—suggests that clarifying accountability under Kazakhstan’s new AI law may matter for adoption alongside technical readiness.

**Abstract:**

**Background/Objectives:** Clinicians’ expectations of artificial intelligence (AI) for periodontal care in Central Asia are largely unknown. We surveyed dentists and physicians in Kazakhstan on preliminary requirements for a future decision-support system and explored patients’ self-reported periodontal signs. **Methods:** Cross-sectional online surveys (July–September 2026; non-probability recruitment) included 33 dentists, 91 primary health care physicians and medical specialists, and 455 adult patients whose periodontal eligibility was not clinically verified; the patient questionnaire did not address AI. Analyses used Wilson 95% confidence intervals (CI), exact tests with Holm adjustment and exploratory logistic regression. **Results:** Dentists prioritised history-taking (81.8%) and early gingivitis diagnosis (69.7%). Autonomy choices were spread across five clinician-controlled configurations (low autonomy most frequent, 30.3%); no autonomous option was offered. Lack of a legal framework and liability insurance was the most cited barrier (60.6%). Physicians and specialists selected early gingivitis screening (80.2%) but cited insufficient competence (53.8%). Self-reported signs occurred in 49.0% of patients. Associations with high stress (adjusted odds ratio 2.11; 95% CI 1.14–3.89) and family history (1.61; 1.02–2.55) lost significance after excluding 204 responses collected via an inter-university platform. **Conclusions:** Clinician responses suggest preliminary requirements; patient associations remain exploratory. Instrument and clinical outcome validation should precede system design.

## 1. Introduction

### 1.1. The Real-World Problem

Periodontal diseases are among the most prevalent chronic conditions worldwide: the World Health Organization (WHO) estimated that severe periodontal disease affects about one billion people [1], and the Global Burden of Disease Study 2021 projected a 40% increase in the number of cases by 2040, driven by population growth and ageing [2]. Periodontitis is bidirectionally linked with diabetes mellitus and independently associated with cardiovascular disease [3,4,5], which is why the 2023 joint workshop of the European Federation of Periodontology (EFP) and the European arm of the World Organization of Family Doctors (WONCA) called on family physicians to screen for periodontal disease and to refer patients with diabetes, cardiovascular and respiratory diseases to dental care [6], in line with the WHO Global Oral Health Action Plan 2023–2030, which prioritises the integration of oral health into primary health care (PHC) and the use of digital technologies [7]. Kazakhstan illustrates the distance between this agenda and current practice. WHO reported a prevalence of severe periodontal disease of 15.3% among people aged 15 years and older, a dentist density of 2.9 per 10,000 population and the absence of a national oral health policy or action plan as of 2021 [8]; the age-standardised prevalence of periodontal disease in Central Asia was estimated at 9321 per 100,000 population in 2021 [2]; and in a clinic-based study of 642 adults attending dental institutions in Astana the prevalence of gingivitis and periodontitis reached 99% and 69% in the 66–80-year age group [9]. Although PHC in Kazakhstan has been reorganised around general practitioners over the past decade, in the spirit of the Astana Declaration adopted in the country’s capital in 2018 [10], to our knowledge no data exist on whether physicians screen for or refer periodontal disease, on the referral pathways available to them, or on what tools they would need to take on this role.

Internationally, integration of oral health into medical care is hampered by limited training of physicians in oral examination, time constraints, unclear referral pathways and the absence of simple screening tools [11]; even where the diabetes–periodontitis link is widely acknowledged, as among Swiss endocrinologists and general practitioners, referral to a dentist remains infrequent [12], and oral-health recommendations in diabetes guidelines are rarely implemented in primary care [13]. These are precisely the gaps in periodontal screening and referral that a decision-support tool for PHC would have to address.

### 1.2. Current State of the Art

Artificial intelligence (AI) has been proposed as a way to close this diagnostic and interprofessional gap. In development studies, deep-learning models have detected periodontal bone loss on panoramic radiographs with clinician-level accuracy [14], staged periodontitis automatically [15], and detected gingival inflammation on standardised intraoral photographs, with pixel-level sensitivity of 0.92 and specificity of 0.94 in a held-out validation set of images [16]; a pilot external validation of the same tool among older adults attending community daycare centres reported lower specificity than the development study [17]. These performance figures should be distinguished from validated clinical applications: they derive predominantly from retrospective, often single-centre studies on curated images, few models have been tested prospectively, performance typically falls on external validation, and, to our knowledge, no periodontal AI model has yet been shown in a prospective clinical study to improve diagnostic accuracy in routine practice, screening outcomes or patient care [18,19,20,21]. Reported diagnostic accuracy therefore does not demonstrate clinical effectiveness or readiness for routine implementation. Scoping and systematic reviews confirm that most of the evidence base concerns radiographic image analysis, with far fewer studies on clinical decision support, risk prediction from systemic data or remote monitoring [18,19]. A recent systematic scoping review with evidence from Kazakhstan identified two dominant paradigms—image-based deep learning for radiographic bone-loss detection and machine-learning-based non-clinical screening using patient-reported data and salivary biomarkers—documented a consistent drop in performance on external validation, and proposed a phased implementation model for emerging health systems that ends with legal governance covering liability and insurance [20]. Schwendicke et al. emphasised that the “chances” of dental AI will only materialise if problems of data quality, external validation, explainability, regulation and workflow integration are solved [21], and a checklist for the reporting of dental AI research was proposed to raise methodological standards [22]. Ethical analyses have mapped issues of accountability, transparency, autonomy and justice specific to dental AI [23,24], while a framework for medical AI describes a continuum of autonomy levels from human-in-the-loop decision support to fully autonomous systems, each requiring different regulatory safeguards [25]. Explainability, trust and clear allocation of liability are consistently identified as preconditions for clinician acceptance of AI in health care [26,27,28,29].

Empirical evidence on stakeholders’ expectations has accumulated rapidly since 2020, almost exclusively through cross-sectional online questionnaires analysed with descriptive statistics, χ^2^ or Fisher’s exact tests, non-parametric comparisons and, increasingly, ordinal or logistic regression [30]. A survey of 155 Australian dentists and dental students found that 91.6% viewed AI as an assistant to the clinician, with concerns focused on accuracy, insurance liability and lack of transparency [31]. In Bulgaria, 54.7% of 53 dentists preferred clinician-guided AI diagnosis and only 3.8% trusted a fully automated diagnosis [32]. In Kuwait, 300 dental professionals named lack of training (71.2%), technical challenges (63.4%) and cost (56.5%) as the main barriers [33]; in Poland, 183 dentists showed moderate acceptance but almost unanimously rejected AI replacing the dentist [34]; a multi-country survey documented that AI use rises with educational level [35]; and a multicentre study of Egyptian periodontists identified educational gaps as the main determinant of adoption [36]. Questionnaire studies specifically in periodontology report high interest but limited knowledge of AI among dentists in India [37,38]. A multidisciplinary survey in Romania that included dentists, legal professionals and forensic physicians showed that 87.8% would not trust a recommendation whose reasoning could not be verified and 74.0% would refrain from using AI while legal liability remained unclear [39]. Patients, in turn, hold generally positive attitudes towards dental AI but expect the dentist to remain responsible for the decision [40].

### 1.3. Research Gap

Three gaps are apparent. First, all of the studies cited above originate from high- or upper-middle-income countries in Europe, the Gulf, South Asia and Australia; no study has examined stakeholder expectations of periodontal AI in Central Asia, a region with a high periodontal burden, a comparatively low dentist density and a distinct post-Soviet organisation of dental and medical services. Second, the existing surveys are almost exclusively single-stakeholder: they describe dentists’ attitudes, or patients’ attitudes, but rarely examine dental and medical clinicians within one study, even though an AI system intended to bridge dental and medical care must serve both professions. Third, previous work has rarely connected clinicians’ declared data requirements (which comorbidities and risk factors an algorithm should use) with patient-level data on whether those variables are associated with periodontal signs in the target population. The contribution of the present study is accordingly descriptive and contextual rather than methodological: it does not propose a new approach to AI development, but provides a simultaneous survey of dentists and physicians in Kazakhstan about a future AI tool, complemented by a patient survey that did not ask about AI and serves only as an exploratory source of data on candidate algorithm inputs, at a moment when the regulatory context has changed. Kazakhstan adopted Central Asia’s first Law “On Artificial Intelligence” (No. 230-VIII of 17 November 2025), which introduces risk-based classification, transparency obligations and mandatory notification of users interacting with AI [41,42], in parallel with the risk-based approach of the European Union AI Act [43] and the WHO guidance on ethics and governance of AI for health [44].

### 1.4. Objectives

The study had the following objectives: (1) to describe which diagnostic procedures, data inputs, output formats and clinical stages the responding dentists prioritise for an AI-enabled periodontal decision-support system, how their choices were distributed among the clinician-controlled configurations offered in a question framed in terms of legal permissibility, and which barriers they perceive; (2) to describe how PHC physicians and medical specialists view their role in periodontal screening, which patient groups they prioritise for AI-assisted screening and which barriers they perceive; (3) to compare the two clinician groups on the few related items that permit cautious comparison; (4) to describe self-reported periodontal signs and their exploratory associations with behavioural and systemic factors among adult patient respondents, whose diagnostic eligibility was not clinically verified, and to examine whether the symptom items form a reliable summed scale; and (5) to distinguish directly measured clinician responses from exploratory patient associations and author-derived proposals for future requirements, all requiring independent validation before system design or governance decisions.

## 2. Materials and Methods

### 2.1. Study Design and Reporting

We conducted a cross-sectional, questionnaire-based study with three parallel target populations. The design follows the approach used in recent stakeholder surveys on dental AI [33,34,39], which we chose because the research questions concern the distribution of preferences and their association with respondent characteristics at a single time point, for which a cross-sectional survey is the appropriate and most resource-efficient design. The three questionnaires were developed together as components of a single stakeholder assessment with a shared conceptual framework (clinical stages of periodontal care, comorbid risk factors, data inputs and barriers to implementation), but each was written for its own respondent group and administered as a separate form; they were not designed as parallel instruments with identical items. Only two content areas were intended for direct comparison between dentists and physicians: the list of seven comorbid conditions, which used largely identical option labels but different item stems (conditions an AI algorithm should take into account, for dentists; conditions that should trigger an automatic AI alert about the risk of tooth loss, for physicians) and one differently worded option (hormonal disorders vs. hormonal changes), and the stage of periodontal care at which AI (dentists) or PHC involvement (physicians) is most useful, for which the item stems and most options differed and only two options were semantically comparable (Section 3.4). Between-group comparisons are restricted to these items and are interpreted with this caveat. Reporting follows the Consensus-Based Checklist for Reporting of Survey Studies (CROSS) [45] and, for the web-based data collection, the Checklist for Reporting Results of Internet E-Surveys (CHERRIES) [46]; the completed CROSS checklist is provided as Supplementary Material S1. The observational elements of the analysis (associations between behavioural and systemic factors and self-reported signs) are reported in accordance with the STROBE recommendations for cross-sectional studies [47].

### 2.2. Setting and Participants

The study was coordinated by Astana Medical University, Kazakhstan, and targeted practising dentists, PHC physicians and medical specialists of somatic profile, and patients invited through dental or PHC facilities. Recruitment was non-probabilistic and used several channels. The dentist link was circulated in a professional messenger chat of dentists in Astana and to private clinics collaborating with the first author’s dental practice, which explains the predominance of private practitioners from Astana. The physician link was forwarded through professional associations, clinic administrations and University clinical bases in Astana and other regions. Patients were intended to be invited by treating clinicians; in addition, the links were disseminated through the messenger channels of the working group’s partners within the inter-university discussion platform of the Center of Epidemiology and Management, Astana Medical University, which reaches partner medical universities. Dissemination through this platform was most intensive at the start of the academic year in early September, after the summer vacation period (Section 3.1). The original Russian participant-information materials specify more than five years of practice for dentists and, for patients, age > 20 years and a diagnosis labelled K05.4 (periodontosis) (Supplementary Material S6). The diagnosis should not be retrospectively broadened to all K05 conditions: the coded survey data contain neither a diagnosis code nor clinical examination findings, the diagnosis date or current treatment status. Although the patient information sheet envisaged extraction of periodontal-chart data, no linked clinical-record data were available for this survey analysis. Diagnostic eligibility and the distribution of actual diagnoses therefore could not be verified. The source of each invitation was not recorded, which also prevents verification of the clinical recruitment pathway for individual anonymous responses. In the achieved sample, 15 dentists had ≤5 years of experience and 82 patients were aged 18–20 years; 76 of these patients belonged to the September response block described in Section 3.1. The protocol thresholds had not been enforced in the electronic forms. During analysis, these practising clinicians and adult patients were retained under broader achieved-sample criteria rather than excluded retrospectively, to describe all collected responses and examine the consequences of eligibility deviations transparently. This was a post-collection departure from the stated thresholds, not evidence that the thresholds were optional in the original materials. Results are reported by dentists’ experience, and a patient model restricted to age > 20 years is provided (Section 2.8). The findings describe the responding groups and do not constitute estimates for a protocol-confirmed disease cohort. Because links were forwarded, invitation denominators and response rates are unknown. The small dentist sample, predominantly private practitioners in Astana, and the physician sample are not representative of their professions in Kazakhstan. Participation was voluntary and anonymous.

### 2.3. Instruments

Three structured questionnaires were developed in Russian by a working group of periodontologists, PHC physicians, epidemiologists and a medical informatics specialist, drawing on published surveys [31,32] and the EFP/WONCA consensus domains [6]. The item pool was reviewed in two rounds for relevance, clarity and completeness by the members of the working group themselves (periodontologists, PHC physicians, epidemiologists and a medical informatics specialist); no external expert panel was convened, revisions were agreed by consensus without a quantitative content-validity index, and individual ratings were not recorded. The Kazakh version was produced by forward translation from Russian by the doctoral candidate (Y.A.) and was submitted to the Local Bioethics Committee together with the protocol and approved; no back-translation, independent bilingual review or cognitive testing in Kazakh was performed, so no formal translation or cross-cultural adaptation procedure can be claimed. A pilot with five clinicians and ten patients examined comprehension and completion time (10–15 min). These steps provide preliminary face and content validity only; no test–retest, construct or criterion validation was undertaken. Comprehension was not evaluated separately by language. The Russian paper versions of the three questionnaires, each preceded by a signature-based informed-consent form, were approved by the Local Bioethics Committee and are reproduced in Supplementary Material S6 together with the participant-information documents and their English translation; in the electronic versions used for data collection (S5), the study information, consent statements and questionnaire were combined in a single form. The post-collection Google Forms printouts (S5) display Kazakh and Russian text together for the information screens and thematic questions, with some demographic options, such as sex for clinicians, in Russian only. The authors’ comparison of the original exports described predominantly Russian response labels with a few bilingual variants; this differs from the more extensively bilingual post-collection printouts. The language relied on by respondents was not recorded, no dated form-change log was available for verification, and the printouts cannot establish the wording and settings at every point during collection. No language-equivalence validation can therefore be claimed. The dentist questionnaire had 15 closed thematic items and five open-ended questions on automation, clinical and laboratory inputs, indices difficult to interpret, stages of care, functions, outputs, transparency, treatment criteria and barriers. Its autonomy question asked what configuration was considered permissible for diagnosing periodontitis under Kazakhstan’s legislation. It offered five single-choice configurations: low autonomy; medium autonomy; mandatory clinician verification; a second opinion; and prohibition of full autonomy. Every option retained clinician control, so the response distribution cannot establish acceptance or rejection of autonomous AI. The options mix technical scope, verification arrangements and a normative position, and the legal framing also mixes perceived legal permissibility with personal attitudes; the item is neither an ordinal scale nor a validated measure of preferred autonomy. The physician questionnaire had ten closed thematic items (Q4–Q13), respondent-characteristic items and three open-ended questions. It covered diabetes-related criticality, systemic factors, referral, screening, barriers, quality-of-life indicators and AI-screening priorities. The patient questionnaire had 20 thematic items covering symptoms, oral hygiene, smoking, stress, diet, attendance, previous treatment, family history, glycaemic status, cardiovascular and musculoskeletal conditions and xerostomia. Five items intended as a single rating or mutually exclusive choice contained multiple selections (Section 2.5). S2 provides the analysis coding and an English translation; S5 preserves the post-collection form printouts without altering their content, while Table S6 distinguishes observed response-type problems, differences between the approved paper versions and the online forms, and uncertainties about historical versions.

### 2.4. Data Collection

Questionnaires were implemented in Google Forms and were open from 27 July to 19 September 2026. IP addresses and e-mail addresses were not collected, sign-in was not required, and single participation was not technically guaranteed. Invitations were forwarded through professional channels, treating clinicians and the partner channels of the inter-university discussion platform (Section 2.2) rather than posted as an unrestricted public recruitment campaign, although the links themselves could be shared. The documented information screens explain voluntary participation and display four consent statements before the questionnaire. The supplied coded analytical datasets do not retain the item-level consent selections or a validation-settings log; a required checkbox question alone does not establish that all four statements were technically enforced for every historical submission. The account of electronic consent is therefore limited to the documented process rather than individual verification of all four selections. After export, records were screened for exact and near-duplicate response patterns and submission timestamps were examined (Section 2.5 and Section 3.1). Professional status was self-declared; clinical eligibility and the individual recruiting source were not linked to the anonymous data. Closed items had recorded answers in the cleaned files, with uncertainty represented by explicit options. For the dentists’ five open questions, only 30, 27, 21, 25 and 29 substantive answers respectively were retained, although the later printouts mark these questions required. The available materials do not resolve whether this discrepancy reflects earlier settings or preparation of the open-answer file, so historical equivalence of required-item settings is not assumed.

### 2.5. Data Processing

**Figure 1:**
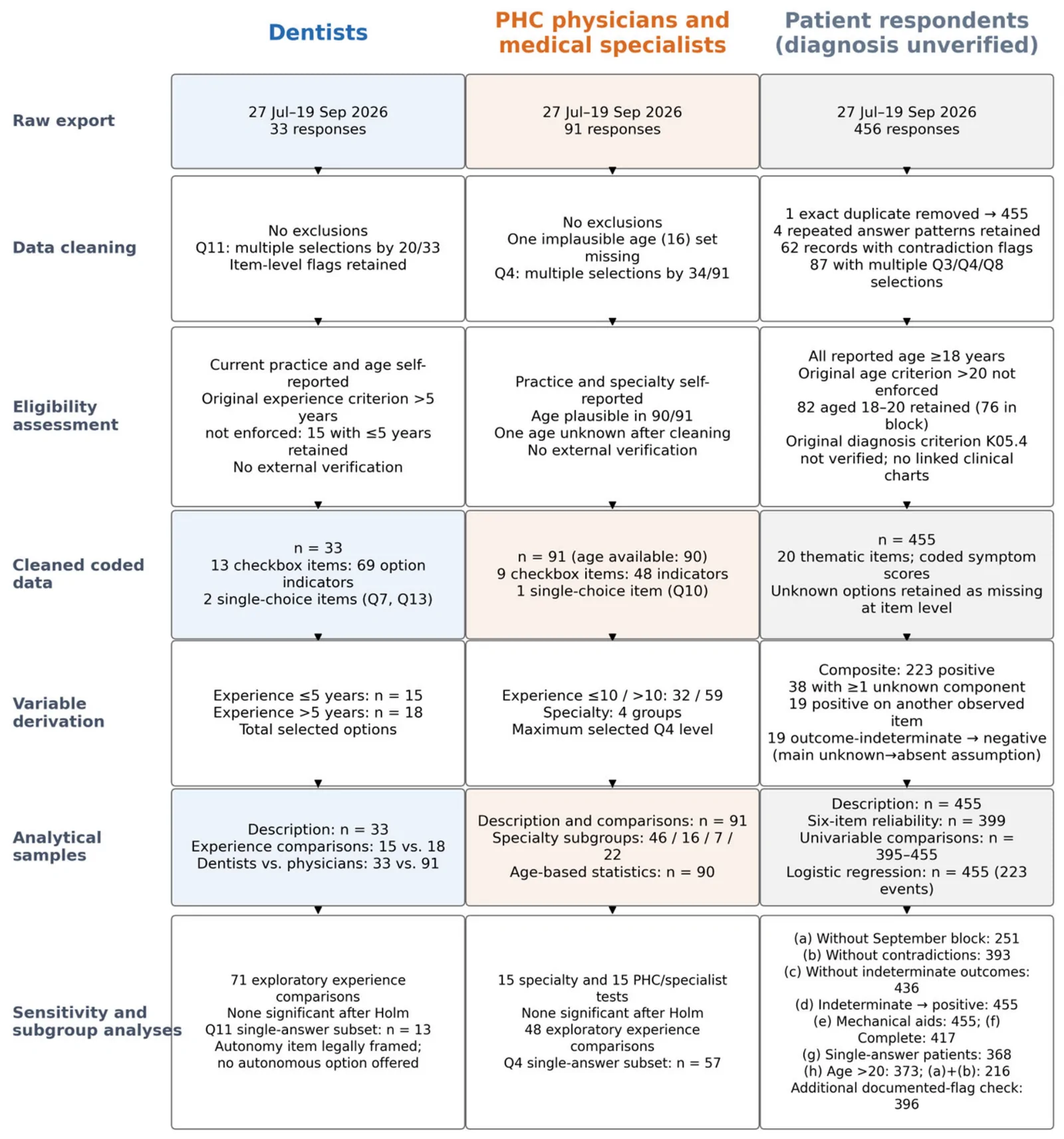
Data-processing flowchart from the raw questionnaire exports to the analytical samples for the three surveys, distinguishing data cleaning from eligibility checks (which relied on self-reported answers and were not externally verified), showing the handling of unknown components of the patient composite outcome, and listing the sensitivity and subgroup analyses. PHC, primary health care.

Figure 1 summarises the transition from the raw exports to the analytical samples, distinguishing data cleaning from the verification of eligibility. Exports were checked for completeness, exact duplicates, near-duplicates, logical contradictions and out-of-range values. One exact duplicate was removed from the patient file (456 → 455 records). The exact-duplicate pair identified by the authors was submitted on 2 September, 43 min apart. No two of the retained records had an identical response pattern across all coded items; after age and sex were disregarded, four repeated response patterns were identified, involving 13 records (one pattern shared by seven respondents and three patterns shared by two respondents each). Most symptom responses in these patterns indicated no current symptoms, but the patterns did not consist uniformly of first response options (for example, 11 of the 13 records gave the last option of the symptom-duration item, “there were no such symptoms”). These records were retained; their recurrence establishes neither duplicate participation nor that they came from different respondents. In the physician file, one respondent’s stated age of 16 years was judged impossible for a practising physician and set to missing (age available for 90 of 91). No records were excluded from the dentist file (*n* = 33). Each response option of a multiple-choice item was converted into a dichotomous indicator (0 = not selected, 1 = selected); percentages were calculated from the number of respondents and therefore may sum to more than 100% within an item. In five items that were intended as mutually exclusive choices or a single rating, according to the approved paper versions (S6), the online forms used checkboxes and the exported responses contained multiple selections: the dentists’ item on the frequency of risk re-assessment (Q11; 20 of 33 respondents selected more than one option), the physicians’ ordinal item on the importance of periodontal status for diabetes control (Q4; 34 of 91), and the patients’ items on tooth mobility (Q3; 10 of 455), visual gingival changes (Q4; 39) and additional hygiene aids (Q8; 47); 87 patients selected more than one option in at least one of these three items. In the later form printouts (Supplementary Material S5), the physicians’ Q4 and the three patient items carried the instruction “you may select several options”, whereas the dentists’ Q11 lacked that extra wording in its stem but displayed the standard form instruction to check all that apply. Its options also referred to different settings: twice yearly without somatic disease, four times yearly with diabetes or cardiovascular disease, monthly through an app, annually at routine examination, or at exacerbation. Multiple selections may therefore partly reflect different clinical contexts and do not identify one preferred frequency. In the approved physician paper version (S6), no item was marked as multiple-choice; online, all thematic physician items except Q10 were checkbox items with this instruction, and multiple selections occurred in each of them (34 to 65 of 91 respondents per item). All other items intended as single-choice (dentists’ Q7 and Q13, physicians’ Q10 and the remaining 17 patient items) were radio-button items online, and every respondent gave exactly one answer to them (Table S6). The original submission stated that six single-choice items were affected; this figure was an error, as both the forms and the export show five. Two options of the approved dentist paper version (S6; “all signs in combination” in Q2 and “all indicators over time” in Q8) are absent from the later printouts and appear in the coded data only as all-zero placeholder indicators (D2_6 and D8_5), carried over from the paper-version coding scheme. They were not analysed as zero-frequency options; their availability throughout collection cannot be established from the later form state. The physician diabetes option is present in the printout and coded data but was absent from the approved paper version (S6; Table S6). The affected items were analysed as multiple-choice items (each option as a 0/1 indicator); for the two ordinal items the maximum selected level was additionally derived (mobility: highest degree selected; physicians’ Q4: highest importance level selected), and the hygiene item was dichotomised as described in Section 2.6. The consequences are that the percentages for these items are not mutually exclusive, that the physicians’ ordinal item cannot be treated as a single rating (46.2% selected “extremely critical”, but only 16 of these 42 respondents selected it as their sole answer), and that statistics based on the maximum selected level assume that the highest selected category best represents the respondent’s view, an assumption that could not be verified. To examine its consequences, the affected items were also analysed after excluding respondents who selected more than one option: dentists’ Q11 (13 single answers), physicians’ Q4 (57 single answers) and, in the patient regression, the 87 patients with multiple selections in Q3, Q4 or Q8 (Section 2.8; Table S11). Internal inconsistencies in patient records were flagged but retained, because they could not be resolved without re-contacting anonymous respondents: “teeth are firm” together with a reported degree of mobility (6 records), “gingiva pale pink and firm” together with a pathological gingival sign (23), “additional hygiene aids are unnecessary” together with any other option of the item, including “very rarely” (3), “no previous treatment” together with one or more treatment courses per year (12), “never had symptoms” together with curettage or flap surgery (17), and a dispensary-registration answer judged inconsistent with the respondent’s other answers during the original data cleaning (5; according to the codebook, this flag marks a mismatch between the dispensary-registration answer and the diagnosis entered in the following free-text item (“if registered, please specify the diagnosis or diagnoses”; Supplementary Materials S5 and S6); because that free-text field was not retained in the coded dataset, the flag cannot be re-derived from the coded variables and is used as stored, and disregarding it changed analysis (b) only marginally; Supplementary Material S3); 62 records had at least one contradiction. For the composite outcome, a contradiction was resolved in favour of the reported sign (the highest selected mobility degree and the count of pathological gingival signs were used), so contradictory records were more often classified as positive (42 of 62, 67.7%) than the remaining records (181 of 393, 46.1%); the effect of excluding them was examined in a sensitivity analysis (Section 3.6). Cleaned datasets were stored in SPSS-compatible syntax executed in GNU PSPP, version 2.0.X (Free Software Foundation, Boston, MA, USA, 202X) format; the codebooks, contradiction and multiple-selection flags and all derivation rules are provided in Supplementary Material S3, and the analysis code in Supplementary Material S4, which re-derives 65 stored variables from the coded item responses and checks them against the stored values; the stored variables that cannot be re-derived from the coded files (the dispensary-status contradiction flag, the response-block indicator and the clinicians’ specialty group) are listed in the code and defined in Supplementary Material S3.

### 2.6. Variables

Ordinal patient responses were scored in increasing order of severity (bleeding 0–5, halitosis 0–4, mobility 0–4, number of pathological gingival signs 0–5, pain 0–5, suppuration 0–4, xerostomia 0–4, stress 0–5, symptom duration 0–5); toothbrushing frequency and dental attendance were reverse-coded so that a higher score denoted a less favourable value throughout. “Cannot say” responses were treated as missing (≤7.5% for any item) in item-level analyses and were not imputed; their handling in the composite outcome is described below. Binary risk-factor indicators were derived as follows: current smoking (any current tobacco use, including heated tobacco), toothbrushing less than twice daily, use of adjunctive hygiene aids, high stress (self-rated high, chronic or depressive level), dysglycaemia (self-reported prediabetes or diabetes; respondents who had never had their glucose measured were kept as a separate category), cardiovascular disease (any reported diagnosis), positive family history of periodontitis or early tooth loss (unknown history kept as a separate category), and dental attendance at least once a year. The adjunctive-hygiene-aid indicator used in the original analysis was defined as selection of any of “the full complex daily”, “floss only”, “mouthwash only” or “interdental brushes as recommended” (277 patients, 60.9%); because these options specify different frequencies (“daily”, “as recommended” or none), the indicator records use of an aid rather than a common frequency of regular use. Because mouthwash is not a mechanical interdental cleaning aid, a narrower indicator of mechanical interdental cleaning (full complex, floss or interdental brushes; 242 patients, 53.2%; 35 patients used mouthwash only) was defined during revision and substituted in a sensitivity analysis. Professional experience was dichotomised at 5 years for dentists (the protocol’s inclusion threshold) and at 10 years for physicians (the median category). The primary patient outcome was a composite indicator of self-reported signs of periodontal disease, defined as at least one of four components, each derived from one questionnaire item (Table S2-C): gingival bleeding at least when eating hard food (Q1, score ≥ 2); any tooth mobility (Q3, any of the four mobility degrees selected); at least one pathological gingival appearance (Q4, any of “redness, swelling”, “gaps between the teeth”, “bluish tint”, “pustules/swellings” or “bright red band”); or suppuration at least “sometimes on pressure” (Q14, score ≥ 2). This composite is a pragmatic, study-specific outcome: it combines non-specific self-reported symptoms, it is not a validated instrument, and it is not equivalent to clinically diagnosed periodontitis; it is referred to throughout as “self-reported periodontal signs”. The choice of components was informed by the CDC/American Academy of Periodontology self-report approach [48], which, however, used a different set of questions, combined them with demographic variables and was validated against clinical periodontal examination; neither our items nor our thresholds have been validated in this way. The bleeding threshold (“at least when eating hard food”) deliberately excluded the response “very rarely”, chosen by 51.2% of patients, to avoid classifying trivial bleeding as a sign of disease; this threshold is the reason why 71.4% of patients reported any bleeding whereas 49.0% met the composite (Section 3.5). In total, 38 patients had at least one unknown component: “cannot say” for suppuration (*n* = 25) or the sole response “teeth absent or replaced by complete dentures” for mobility (*n* = 14), one patient having both. In the main analysis an unknown component was assumed to indicate absence of the sign; this is a single deterministic assumption (equivalent to imputing “absent”), not a complete-case analysis. Under this assumption 19 of the 38 patients were classifiable regardless, because they reported another positive component, whereas the remaining 19 had no observed positive component and were classified as negative although their true status is indeterminate. The assumption was examined in three sensitivity analyses: exclusion of the 19 outcome-indeterminate records, their reclassification as positive, and restriction to the 417 patients with all four components known (Section 2.8).

### 2.7. Sample Size Considerations

No formal a priori sample size calculation was performed for the exploratory clinician surveys; all retained responses received during the study period were analysed. Estimates from 33 dentists are imprecise: the Wilson 95% confidence intervals reported with every proportion have half-widths of about ±17 percentage points for *n* = 33 and ±10 points for *n* = 91 at *p* = 50%, and the comparison of 15 with 18 dentists has low power for anything but large differences. Between-group comparisons in these samples are therefore hypothesis-generating, and the absence of a statistically significant difference is reported as “not demonstrated” rather than as evidence of agreement between groups. For the patient survey, the 223 outcome events allowed a logistic model with 12 predictor terms while maintaining 18.6 events per variable, above the conventional minimum of 10 [49]. Because recruitment was non-probabilistic, no sampling-based margin of error is reported for the patient proportions; the confidence intervals describe statistical precision conditional on the sample obtained, not its representativeness.

### 2.8. Statistical Analysis

The analytical strategy was chosen to match the measurement level of the data and the small size of the clinician samples, following the practice of comparable published surveys [33,34,39]. Categorical variables are presented as *n* (%) with 95% confidence intervals (CI) calculated by the Wilson score method, which retains nominal coverage for small samples and proportions near 0 or 100% [50]. The distribution of quantitative variables was assessed with the Shapiro–Wilk test, skewness, kurtosis, histograms and normal Q–Q plots; because no quantitative variable was normally distributed, they are summarised as median(first quartile–third quartile), Me (Q1–Q3),and compared with rank-based methods. Two independent groups were compared with the Mann–Whitney U test (asymptotic), with the effect size r = Z/√N and its 95% CI obtained by percentile bootstrap (1000 resamples); three or more groups were compared with the Kruskal–Wallis H test with ε^2^ as effect size. Categorical variables were compared with Pearson’s χ^2^ test; when any expected frequency was below 5, Fisher’s exact test (2 × 2 tables) or its Freeman–Halton extension (2 × k tables) was used, with φ, Cramér’s V and odds ratios (OR) with Woolf 95% CI (0.5 added to all cells in the presence of a zero cell) as effect sizes. Associations between ordinal variables were quantified by Spearman’s rank correlation coefficient (ρ). The internal consistency of the six patient symptom items was examined with Cronbach’s α (95% CI by Feldt’s method), corrected item–total correlations and α-if-item-deleted, with α ≥ 0.70 as the criterion for combining items into a scale. Factors associated with the composite indicator of self-reported periodontal signs were first examined in univariable analyses and then in a multivariable binary logistic regression with simultaneous entry of the following predictors (age per 10 years, sex, smoking status, toothbrushing frequency, use of adjunctive hygiene aids, high stress, glycaemic status, cardiovascular disease and family history), categorical predictors being indicator-coded. Model performance was described by the likelihood-ratio χ^2^, Cox–Snell, Nagelkerke and McFadden R^2^, and the area under the receiver operating characteristic curve (AUC); multicollinearity was checked with variance inflation factors (VIF). The AUC is the apparent (in-sample) value; no internal (bootstrap or cross-validation) or external validation was performed, so it describes the fit of the model to this dataset and not its performance as a clinical risk-prediction tool, for which the model is not intended. Sensitivity analyses of the regression were introduced at two stages, which we distinguish explicitly; no dated statistical analysis plan specified them in advance. Analysis (a), exclusion of a block of 204 responses received on 2–4 September 2026 with an atypical age structure (190 of them aged 20–22 or 42 years), was specified after inspection of the data and before the regression model was fitted. Analyses (b)–(h) were added during peer review: (b) exclusion of the 62 records with internal contradictions; (c) exclusion of the 19 outcome-indeterminate records (no observed positive component and at least one unknown component); (d) reclassification of these 19 records as positive; (e) replacement of the adjunctive-hygiene-aid indicator by the mechanical-interdental-cleaning indicator; (f) restriction to the 417 patients with all four composite components known; (g) exclusion of the 87 patients who selected more than one option in any of the three patient items affected by multiple selection (Q3, Q4, Q8); and (h) restriction to patients aged >20 years, the age criterion of the original protocol (*n* = 373). A combined exclusion of (a) and (b) was also examined, and the composite frequency, the univariable associations and the reliability analysis were repeated without the response block. For physicians, the principal findings were tabulated by specialty group (general practitioners/internists, paediatricians, endocrinologists, other specialists) and compared across the four groups with the Fisher–Freeman–Halton exact test and between PHC physicians and medical specialists with Fisher’s exact test. To guard against over-interpretation of the large number of exploratory comparisons, the number of nominally significant results in each family of tests was compared with the number expected under the global null hypothesis (5% of tests), and Holm–Bonferroni-adjusted *p* values [51] were calculated for each family (71 experience comparisons among dentists: 69 response options and two single-choice items; 48 experience comparisons among physicians; 15 four-group and 15 two-group specialty comparisons among physicians; nine dentist–physician comparisons; 56 factor–symptom comparisons, eight univariable factor comparisons, 14 comparisons of symptom scores by age group and smoking status and 66 Spearman correlations among patients); all subgroup results are interpreted as hypotheses rather than confirmatory findings. The remaining tests—comparisons of the total number of selected options and of the maximum selected criticality level among clinicians, the age comparison between the response block and the other patients, and the *p* values of individual regression coefficients in the multivariable model and its sensitivity analyses (Section 3.5 and Section 3.6; Supplementary Table S8)—are reported without adjustment (nominal p values) and are used only for exploratory description. The sensitivity analyses were not treated as one family of tests, because they re-estimate the same model under alternative data-handling assumptions rather than test different hypotheses. Two-sided *p* < 0.05 was considered statistically significant and exact *p* values are reported. Open-ended answers were not subjected to a formal qualitative analysis: the dentists’ answers on responsibility for an AI-supported decision were tallied descriptively into three categories (responsibility with the clinician; shared or split responsibility; other or unclassifiable; Table S12), and other open-ended answers are quoted only as illustrative examples, without claims about frequency or consensus. Analyses were performed in Python, version 3 (Python Software Foundation, Wilmington, DE, USA) (pandas, SciPy, statsmodels) and with SPSS syntax executed in GNU PSPP 2.0; key statistics were cross-checked between the two environments. The Python code (Supplementary Material S4) re-derives 65 stored variables from the item codes and checks them against the stored values (the three stored variables that cannot be re-derived are listed in the code), reproduces the results reported in the article and its Supplementary Tables, compares every value printed in the reproduced tables and the statistics quoted in the text (2573 values) with the recomputed values and terminates with an error if any re-derived variable or reported value disagrees; the only random procedure (bootstrap confidence intervals of r) uses a fixed seed. The output of the archived run on the final cleaned datasets is provided with S4, and its derivation and verification reports are reproduced in S3.

### 2.9. Ethics

The study protocol was approved by the Local Bioethics Committee of NJSC “Astana Medical University” (Protocol No. 1/ID:312 of 24 June 2026). The approved protocol covered the anonymous online questionnaires administered through Google Forms, including the collection of self-reported health information from patients, the invitation of patients through their treating clinicians, and the dissemination of the questionnaire links through the inter-university discussion platform of the Center of Epidemiology and Management, Astana Medical University, and the messenger channels of the working group’s partners (Section 2.2). This report concerns the anonymous questionnaire component; no linked clinical charts or examination data were analysed. Original participant-information materials with their English translation and the approved paper questionnaires with consent forms are provided in Supplementary Material S6, and the later electronic information and consent screens in S5. These documents describe intended recruitment and consent procedures; they do not independently verify individual diagnoses, historical form settings, or the approval status of each paper-to-online difference. The achieved-sample departures from the stated age and experience thresholds are reported in Section 2.2. Participants were presented with information on voluntary participation, confidentiality and the right to stop the questionnaire. The analytical files do not permit retrospective confirmation of each consent selection (Section 2.4).

## 3. Results

### 3.1. Characteristics of the Participants

Data from 579 respondents were analysed: 33 dentists, 91 PHC physicians and medical specialists, and 455 patients (Table 1). Dentists were predominantly men (63.6%; 95% CI 46.6–77.8), worked mostly in private clinics (87.9%; 95% CI 72.7–95.2) and in Astana (84.8%); 18 (54.5%) had more than five years of experience. Among physicians, women predominated (86.8%; 95% CI 78.4–92.3); 50.5% were general practitioners/internists, 17.6% paediatricians, 7.7% endocrinologists and 24.2% other specialists. Patients were 67.5% women, 63.3% lived in Astana and 9.2% were on a dispensary register. Age was non-normal in each group (Shapiro–Wilk *p* ≤ 0.011). The block of 204 patient responses from 2–4 September 2026 accounted for 44.8% of the patient sample and had a bimodal age structure: 190 respondents were aged 20–22 or exactly 42, including 75 aged 20 and 75 aged 42. Only 14 of the other 251 patients reported those ages. Block respondents were younger (median (Q1–Q3) 22 (20–42) vs. 44 (36–57) years; (median 22 [20; 42] (20–42) vs. 44 [36; 57] (36–57) years; U for the older remainder = 41,751, equivalent to the smaller U = 9453; *p* < 0.001; r = 0.55, 95% CI 0.48–0.61). Their sex distribution, family history, smoking, dysglycaemia, cardiovascular disease, hygiene-aid use and symptom reporting also differed; the full descriptive comparisons are given in Table S13. Signs were reported by 83/204 (40.7%) in the block and 140/251 (55.8%) outside it; contradiction rates were similar (13.2% vs. 13.9%). The block included 76 of the 82 patients who did not meet the original age criterion > 20 years. The authors’ review of the original submission timestamps recorded 0–6 submissions per day from 19 August to 1 September, then 147, 30 and 28 on 2, 3 and 4 September respectively (205 submissions, including the removed duplicate). Within-block submission intervals had a median of 40 s, and 87 were below 30 s. These are intervals between submissions, not individual completion times; concurrent completion can also produce short intervals. On 4 September, twelve consecutive submissions between 08:36 and 09:00 reported age 42 and four distant cities. The block coincided with the dissemination of the links through the inter-university discussion platform of Astana Medical University to partner medical universities at the start of the academic year, after the summer vacation period, when distribution reached its maximum (Section 2.2). This channel explains the timing and volume of the block, and the concentration at ages 20–22 is compatible with respondents drawn from university communities (students and staff) rather than patients invited by treating clinicians. The institution, the forwarding person, any assistance and device information were not recorded for individual submissions, so neither the clinical eligibility of block respondents nor the reason for the age-42 cluster and the closely spaced submissions can be established. All 204 retained records differ in at least one recorded field; across the 20 thematic items, 198 have unique answer sets and six form three matching pairs, with demographic and/or city differences. Distinct patterns do not verify identity or exclude repeated completion with changed answers. The block was retained under the broader achieved-sample adult criterion, not because all records met the protocol criteria. Its dissemination channel is known, but the individual eligibility of its respondents, any assistance and unique participation remain unverified. The analyses without it assess sensitivity rather than establish an objectively preferred dataset.

### 3.2. Dentists’ Requirements for an AI System

Table 2 summarises the most frequent responses of dentists. The procedures they proposed to automate first were history-taking (81.8%; 95% CI 65.6–91.4) and visual assessment of the gingiva (72.7%; 95% CI 55.8–84.9), and on panoramic radiographs the destruction of the cortical plate of the interdental septa (90.9%; 95% CI 76.4–96.9). Early diagnosis of gingivitis was the stage at which AI was considered most needed (69.7%), followed by differential diagnosis of mild versus moderate periodontitis (60.6%) and screening of healthy individuals (57.6%). Diabetes mellitus (81.8%) and the quality of individual oral hygiene (84.8%) were the systemic and behavioural inputs most often proposed for the algorithm; remote monitoring (66.7%), photograph-based index calculation (60.6%) and prediction of tooth loss (57.6%) were the preferred functions; and a graphical periodontal chart with colour-coded risk zones was the preferred output (66.7%). A reduction in probing depth (75.8%) and stabilisation of the interdental septal height (69.7%) were the most frequently chosen criteria of treatment success.

**Figure 2:**
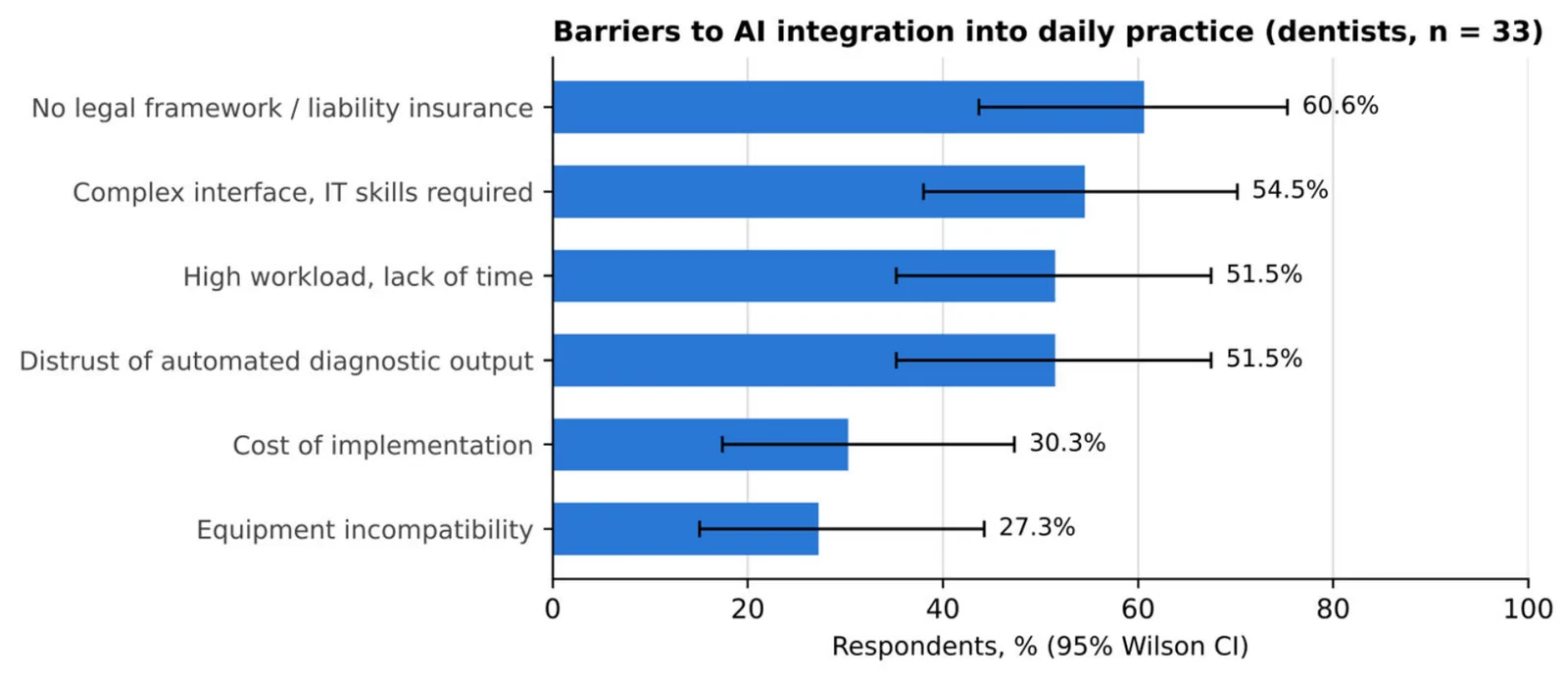
Barriers to the integration of AI into daily practice reported by dentists (*n* = 33): percentage of respondents with 95% Wilson confidence intervals. The distribution of autonomy choices by professional experience is given in Table 3.

Responses to the question on autonomy considered permissible under Kazakhstan legislation were distributed across all five options, none of which was chosen by a majority: low autonomy (AI processes data only; the clinician decides) 10 (30.3%; 95% CI 17.4–47.3), medium autonomy (AI proposes options) 6 (18.2%; 95% CI 8.6–34.4), decision support with mandatory clinician verification 6 (18.2%), AI as a “second opinion” 6 (18.2%) and “full autonomy prohibited, the choice is always the doctor’s” 5 (15.2%; 95% CI 6.7–30.9). Because every option in this item preserved clinician control over the decision, these data describe how choices were distributed among the clinician-controlled configurations offered; they cannot show whether dentists would accept or reject an autonomous system, which was not offered as an option. Of the 29 dentists who answered the open question on liability following discordant AI and clinician diagnoses and a patient complication, 20 placed responsibility on the clinician (for example, “the final decision is always made by the doctor, and AI only helps”), 3 described shared or split responsibility (for example, “50/50”) and 6 gave other or unclassifiable answers (Table S12); this descriptive tally is not a formal qualitative analysis, and the answers do not substitute for the missing autonomous option. The most frequently reported barrier to integrating AI into daily work was the absence of a legal framework and liability insurance (60.6%; 95% CI 43.7–75.3), followed by a complex interface requiring IT skills (54.5%), high workload (51.5%) and distrust of automated diagnostic output (51.5%), whereas equipment incompatibility (27.3%) and cost (30.3%) were cited less often (Figure 2). The confidence intervals of the four leading barriers overlap, so their rank order cannot be established from these data.

The choice of autonomy level differed by professional experience at the nominal level (Table 3). Dentists with ≤5 years of experience more often chose low autonomy (46.7% vs. 16.7%), whereas those with >5 years more often chose decision support with mandatory verification (27.8% vs. 6.7%) or the option “full autonomy prohibited, the choice is always the doctor’s” (27.8% vs. 0.0%) (Fisher–Freeman–Halton exact *p* = 0.045; Cramér’s V = 0.54). The same group more often considered the delimitation of responsibility between clinician and AI to be the most important aspect of transparency (50.0% vs. 6.7%; exact *p* = 0.012; V = 0.60), whereas less experienced dentists prioritised seeing the source data (53.3% vs. 22.2%). Of 69 response options in the multiple-choice items, 7 differed by experience at *p* < 0.05 (Supplementary Table S1), about twice the 3.5 expected by chance; six of them were selected more often by experienced dentists (e.g., crowding as an anatomical risk factor, 94.4% vs. 46.7%; furcation involvement on radiographs, 77.8% vs. 26.7%), and only distrust of automated results was more frequent among less experienced dentists (73.3% vs. 33.3%; OR 0.18; 95% CI 0.04–0.82; *p* = 0.037). The total number of options selected was higher among experienced dentists (Me (Q1–Q3) 43.0 (36.8–51.3) vs. 31.0 (25.5–41.5)) without reaching significance (U = 86.5; *p* = 0.079; r = 0.31). All of these experience-related differences are exploratory: the subgroups comprised only 15 and 18 respondents, 71 comparisons were made, and after Holm–Bonferroni adjustment none remained statistically significant (smallest adjusted *p* = 0.31; Table S1). They are reported as hypotheses for larger studies and should not be read as established differences between professional groups.

### 3.3. Views of PHC Physicians and Medical Specialists

**Figure 3:**
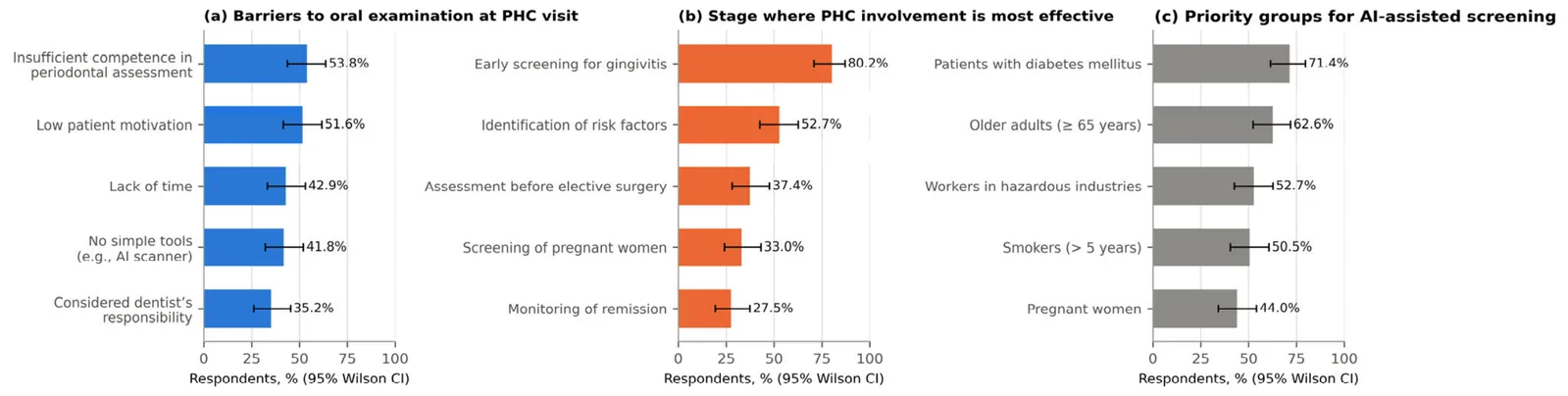
Views of primary health care (PHC) physicians and medical specialists (*n* = 91): (**a**) barriers to oral examination during a medical consultation; (**b**) stage at which PHC involvement in periodontal care is considered most effective; (**c**) priority groups for AI-assisted periodontal screening. Bars show the percentage of respondents with 95% Wilson confidence intervals.

Almost half of the physicians (46.2%; 95% CI 36.3–56.3) selected “extremely critical” when asked how important periodontal status is for the compensation of diabetes (Table 4). This item allowed multiple selections online although it was designed as a single ordinal rating: 34 of 91 respondents selected more than one level, and only 16 of the 42 who selected “extremely critical” selected it as their sole answer, so the percentages overlap and the median of the maximum selected level, 4 [3; 5] (Q1–Q3 3–5) on the five-point scale, should be interpreted cautiously. Among the 57 physicians who selected a single level, “extremely critical” was chosen by 16 (28.1%), “very important” by 17 (29.8%), “important” by 19 (33.3%), “moderately important” by 4 and “of little importance” by 1 (median 4 [3; 5] (Q1–Q3 3–5); Table S11). Severe periodontitis was most often linked to the duration of the underlying systemic disease (75.8%) and the duration of smoking (67.0%); gastrointestinal diseases (67.0%) and diabetes (62.6%) were the comorbidities proposed for automatic AI alerts about the risk of tooth loss; and gingival bleeding was recognised as an early sign by 76.9%. Physicians identified early screening for gingivitis as the stage at which their own contribution would be most effective (80.2%; 95% CI 70.9–87.1) and patients with diabetes as the priority group for AI-assisted screening (71.4%), followed by adults aged ≥65 years (62.6%). The main obstacles to examining the oral cavity during a medical consultation were insufficient competence in periodontal assessment (53.8%), low patient motivation (51.6%), lack of time (42.9%) and the absence of simple tools such as an AI-based scanner (41.8%); 35.2% considered it the dentist’s responsibility (Figure 3). Asked which single indicator would best capture the impact of periodontitis on quality of life and disability-adjusted life years, physicians most often chose emergency visits (33.0%) and economic costs (24.2%).

Because the sample combined general practitioners and specialists with different clinical responsibilities, the principal findings were tabulated by specialty group (Table S7). Early screening for gingivitis was selected as the most effective stage of PHC involvement by 71.7% of general practitioners/internists (*n* = 46), 93.8% of paediatricians (*n* = 16), 85.7% of endocrinologists (*n* = 7) and 86.4% of other specialists (*n* = 22) (Fisher–Freeman–Halton exact *p* = 0.238); patients with diabetes were selected as a priority group by 63.0%, 87.5%, 71.4% and 77.3% (*p* = 0.284); and insufficient competence was named as a barrier by 47.8%, 68.8%, 42.9% and 59.1% (*p* = 0.451). PHC physicians (general practitioners, internists and paediatricians; *n* = 62) and medical specialists (*n* = 29) showed no statistically significant difference on any of the 15 items (Fisher’s exact *p* ≥ 0.167; all Holm-adjusted *p* = 1.000). Across the four groups, four items differed at the nominal level (low patient motivation as a barrier, *p* = 0.009; gastrointestinal disease as an alert condition, *p* = 0.010; smokers as a priority group, *p* = 0.020; and the absence of a simple tool as a barrier, *p* = 0.049), the first three because paediatricians and the fourth because endocrinologists selected them most often; none remained significant after Holm adjustment across the 15 comparisons (smallest adjusted *p* = 0.142). With subgroups as small as seven endocrinologists, the absence of significant differences does not show that the specialty groups hold equivalent views. The maximum selected level of criticality did not differ by specialty (Kruskal–Wallis H(3) = 4.69; *p* = 0.196; ε^2^ = 0.052) or by experience (≤10 vs. >10 years; U = 913.0; *p* = 0.783; r = 0.03), also when the analysis was restricted to the 57 single answers (H(3) = 7.27; *p* = 0.064; U = 350.5; *p* = 0.993), and endocrinologists chose “extremely critical” no more often than other specialists (3/7 vs. 39/84; Fisher’s exact *p* = 1.000). The total number of selected options, however, differed by specialty (H(3) = 13.44; *p* = 0.004; ε^2^ = 0.149): paediatricians selected more options than general practitioners (Me 37 vs. 21; U = 139.5; *p* < 0.001; r = 0.47; 95% CI 0.23–0.66; nominal *p* values) and than other specialists (*p* = 0.004; r = 0.47), which probably reflects response style rather than substantive disagreement, since 9 of 16 paediatricians responded within a single hour on one day. Of 48 response options compared by experience, only one differed at *p* < 0.05 (discomfort on chewing as a sign of gingivitis: 49.2% vs. 25.0%; OR 2.90; 95% CI 1.12–7.49; *p* = 0.028), consistent with the 2.4 chance findings expected and not significant after Holm–Bonferroni adjustment.

### 3.4. Dentists Versus Physicians

Nine items were comparable between the two clinician questionnaires (Table 5; Supplementary Figure S1). The seven comorbid conditions were presented with largely identical option labels but under different item stems (conditions the AI algorithm should take into account, for dentists; conditions for an automatic AI alert about the risk of tooth loss, for physicians), “hormonal disorders” was worded “hormonal changes” for physicians, and the option “diabetes mellitus” was present in the physicians’ online form but not in the approved paper version (S6) (Table S6); these are therefore related rather than identical questions. In addition, the two stage items were taken from questions with different stems (“the stage at which AI is most needed” for dentists; “the stage at which PHC involvement is most effective” for physicians) and different option sets, in which only “early diagnosis of gingivitis”/”early screening for gingivitis” and “monitoring of remission” were semantically equivalent; the stage comparisons therefore compare related but not identical questions. No statistically significant differences were demonstrated for the comorbidities (all *p* ≥ 0.052), the largest discrepancy concerning diabetes, which dentists selected more often (81.8% vs. 62.6%; *p* = 0.052; φ = 0.18). Both groups placed early diagnosis or screening of gingivitis first (69.7% vs. 80.2%; *p* = 0.231), but dentists more often saw a role for AI in monitoring stabilisation during remission (54.5% vs. 27.5%; OR 3.17; 95% CI 1.39–7.23; *p* = 0.010; φ = 0.25), but this difference did not remain significant after Holm adjustment across the nine comparisons (adjusted *p* = 0.087). Given 33 dentists and the unequal group sizes, the power of these comparisons is limited, and the absence of significant differences should not be read as agreement between the professional groups: the confidence intervals of the odds ratios in Table 5 are compatible with both appreciable differences and near-identical views.

### 3.5. Patients: Self-Reported Signs and Associated Factors

Among patient respondents, 71.4% reported any gingival bleeding, but 51.2% selected “very rarely”; 20.2% met the composite bleeding threshold and 15.8% reported bleeding at every brushing or more often. Of those able to answer, 68.0% reported halitosis. At least one pathological gingival appearance was reported by 30.3%, pain by 21.8%, tooth mobility by 20.4% and any suppuration, including a single episode, by 10.3%; 253 respondents (55.6%) stated that they had never had gum-disease symptoms (Table S2). These are symptom reports in the achieved convenience sample, not periodontal-disease prevalence or the symptom frequency of a clinically verified K05.4 cohort. The diagnostic mix and treatment or remission status were not established, so symptom-free reports cannot be explained by presumed previous treatment. The factors examined below are correlates of symptom reporting, not risk factors for developing disease. Behaviourally, 69.9% brushed at least twice daily, 60.9% reported using an adjunctive hygiene aid (53.2% a mechanical interdental aid), 23.3% were current or former smokers and 13.6% reported high stress; 7.0% reported dysglycaemia, 14.1% cardiovascular disease and 28.4% a positive family history.

The six symptom items did not form a reliable composite scale: Cronbach’s α = 0.56 (95% CI 0.48–0.62; standardised α = 0.57; *n* = 399 with complete answers), corrected item–total correlations ranged from 0.19 to 0.38, deleting any item did not raise α above 0.55, and α remained below 0.70 without the 2–4 September block (0.62; 95% CI 0.54–0.70) (Supplementary Table S3). Symptoms were therefore analysed individually and through the four-component binary indicator defined in Section 2.6, which was positive in 223 of 455 patients (49.0%; 95% CI 44.4–53.6; without the 2–4 September block, 140 of 251, 55.8%): 92 patients reported bleeding at least when eating hard food (40 of them with no other component), 93 any tooth mobility, 138 at least one visual pathological gingival sign and 15 suppuration at least on pressure. Had any bleeding, including “very rarely”, been counted, 79.1% would have been positive. The α of the six-item candidate scale refers to summed symptom scores; it is not a measure of the reliability of the binary indicator, which combines four of these items by an “any of” rule and whose reliability was not assessed. The composite is a pragmatic outcome and not a measure of periodontal disease; its rationale is that bleeding, tooth mobility, gingival appearance and suppuration are signs of gingival inflammation or periodontal breakdown that patients can observe, whereas halitosis, pain and xerostomia were considered less specific and were not included; this rationale has not been tested against clinical examination in our population. The low inter-item correlations (Table S3) suggest that the components capture different aspects of symptom experience rather than one construct, which is why no summed score was used and why the composite should be read as “at least one of four signs reported” and nothing more.

**Figure 4:**
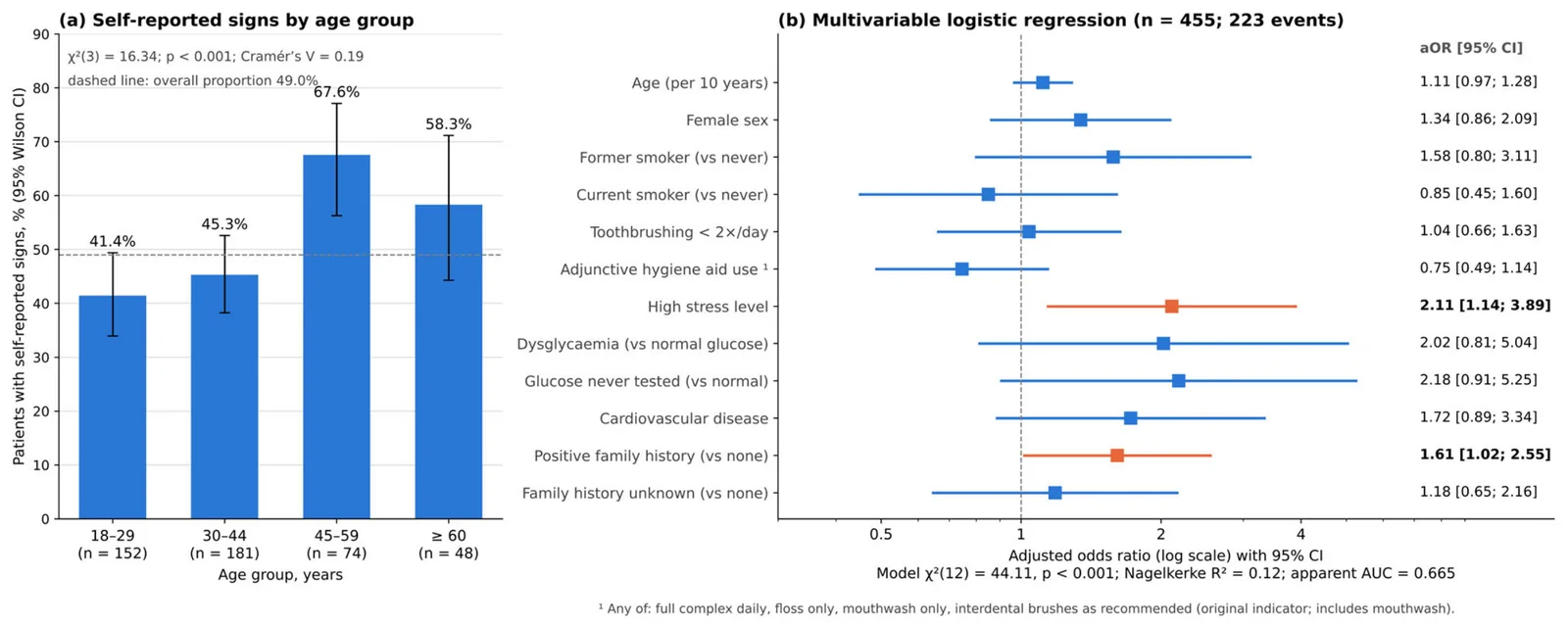
Self-reported signs of periodontal disease in patients (*n* = 455): (**a**) proportion of patients with signs by age group (95% Wilson confidence intervals; dashed line, overall proportion); (**b**) adjusted odds ratios (aOR) with 95% confidence intervals from the multivariable logistic regression (orange, confidence interval excluding 1). The hygiene term is the original adjunctive-hygiene-aid indicator, which includes mouthwash. The AUC shown is the apparent (in-sample) value.

In univariable analyses (Table 6), self-reported signs were more frequent in patients with high stress (69.4% vs. 45.8%; OR 2.68; 95% CI 1.51–4.76), dysglycaemia (75.0% vs. 46.0%; OR 3.52; 95% CI 1.55–8.04), cardiovascular disease (70.3% vs. 45.5%; OR 2.83; 95% CI 1.60–5.02) and a positive family history (61.2% vs. 42.1%; OR 2.17; 95% CI 1.41–3.34), whereas sex, current smoking, brushing frequency and regular dental attendance were not associated with the indicator; all φ coefficients were ≤0.18. These four associations remained significant after Holm–Bonferroni adjustment across the eight comparisons (adjusted *p* = 0.002–0.008). Without the 2–4 September block (*n* = 251), they kept their direction (OR 2.06, 3.46, 2.21 and 1.68, respectively), but none remained significant after Holm adjustment (adjusted *p* ≥ 0.098; Table S10). The proportion with signs rose with age but not monotonically (41.4%, 45.3%, 67.6% and 58.3% in the 18–29, 30–44, 45–59 and ≥60-year groups; χ^2^(3) = 16.34; *p* < 0.001; V = 0.19; Figure 4a). Symptom scores differed by age group or smoking status in 10 of 14 Kruskal–Wallis comparisons at the nominal level and in 5 after Holm adjustment (Supplementary Table S5). At the level of individual symptoms, 27 of 56 factor–symptom comparisons reached *p* < 0.05, always in the direction of higher symptom scores in the presence of the factor, and 12 remained significant after Holm–Bonferroni adjustment (current smoking with halitosis and suppuration; dysglycaemia with gingival signs and xerostomia; cardiovascular disease with mobility, gingival signs and xerostomia; positive family history with halitosis, mobility, pain, suppuration and xerostomia; Supplementary Table S4); a positive family history was associated with all seven symptoms (r = 0.15–0.30; five of them after adjustment) and high stress with six of seven at the nominal level but with none after adjustment, while current smoking was associated with halitosis, pain, suppuration and xerostomia but not with bleeding (r = 0.00; *p* = 0.976). Spearman correlations between symptoms, age and behaviours were all weak (|ρ| ≤ 0.37; 41 of 66 nominally significant and 33 after Holm adjustment; Supplementary Table S9); brushing frequency correlated with dental attendance (ρ = 0.32; *p* < 0.001) but not with bleeding (ρ = 0.08; *p* = 0.099).

In the multivariable model (Table 7; Figure 4b), high stress (adjusted OR 2.11; 95% CI 1.14–3.89; *p* = 0.017) and a positive family history (adjusted OR 1.61; 95% CI 1.02–2.55; *p* = 0.043) remained associated with self-reported signs, whereas dysglycaemia (adjusted OR 2.02; 95% CI 0.81–5.04) and cardiovascular disease (1.72; 0.89–3.34) retained the direction but not the significance of their univariable associations after adjustment for age and the other predictors. The model was significant (χ^2^(12) = 44.11; *p* < 0.001) but explained a small share of the variance (Nagelkerke R^2^ = 0.12) with limited apparent discrimination (AUC = 0.665, in-sample, without validation); VIF ranged from 1.04 to 1.37 and there were 18.6 events per predictor. The two adjusted associations are modest, their lower confidence limits lie close to 1 (1.14 and 1.02), and their *p* values are close to the conventional threshold; together with the limited explanatory performance of the model, this means that the model identifies associations within this dataset and is not suitable for clinical risk prediction without further validation.

**Table 7:** Multivariable binary logistic regression: factors associated with self-reported signs of periodontal disease (patients, *n* = 455; 223 events).

Predictor	B	SE	Wald χ^2^	*p*	Adjusted OR (95% CI)	VIF
Constant	−0.927	0.374	6.13	0.013	0.40 (0.19; 0.82)	–
Age (per 10 years)	0.108	0.073	2.23	0.135	1.11 (0.97; 1.28)	1.21
Female sex	0.294	0.225	1.71	0.192	1.34 (0.86; 2.09)	1.14
Former smoker (vs. never)	0.456	0.346	1.73	0.188	1.58 (0.80; 3.11)	1.12
Current smoker (vs. never)	−0.163	0.324	0.25	0.616	0.85 (0.45; 1.60)	1.19
Toothbrushing < 2 times/day	0.039	0.230	0.03	0.864	1.04 (0.66; 1.63)	1.14
Adjunctive hygiene aid use ^1^	−0.293	0.216	1.83	0.176	0.75 (0.49; 1.14)	1.15
High stress level	0.746	0.313	5.69	0.017	2.11 (1.14; 3.89)	1.09
Dysglycaemia (vs. normal glucose)	0.705	0.465	2.30	0.130	2.02 (0.81; 5.04)	1.24
Glucose never tested (vs. normal)	0.780	0.448	3.03	0.082	2.18 (0.91; 5.25)	1.04
Cardiovascular disease	0.543	0.338	2.58	0.108	1.72 (0.89; 3.34)	1.37
Positive family history (vs. none)	0.476	0.235	4.10	0.043	1.61 (1.02; 2.55)	1.17
Family history unknown (vs. none)	0.168	0.308	0.30	0.586	1.18 (0.65; 2.16)	1.15

Simultaneous entry of predictors; indicator coding with reference categories: male sex, never smoked, normal glucose, no family history. Model: likelihood-ratio χ^2^(12) = 44.11, *p* < 0.001; Cox–Snell R^2^ = 0.092; Nagelkerke R^2^ = 0.123; McFadden R^2^ = 0.070; AUC = 0.665 (apparent, in-sample discrimination without internal or external validation; the model is not intended for individual risk prediction); 18.6 events per predictor. The *p* values of the individual coefficients are nominal (not adjusted for multiple testing) and serve exploratory interpretation. ^1^ Original adjunctive-hygiene-aid indicator: selection of any of “full complex daily”, “floss only”, “mouthwash only” or “interdental brushes as recommended” (Q8; definition in Section 2.6 and Table S2-C); the result obtained with the mechanical-interdental-cleaning indicator is given in Table 8, analysis (e). B, regression coefficient; SE, standard error; VIF, variance inflation factor.

### 3.6. Sensitivity Analysis

Table 8 summarises the sensitivity analyses (adjusted odds ratios for all predictors in Supplementary Table S8). Without the 2–4 September response block (*n* = 251; 140 events), the model remained significant (χ^2^(12) = 25.29; *p* = 0.014; Nagelkerke R^2^ = 0.128; AUC = 0.674) and all effects kept their direction, but no predictor reached significance (high stress: aOR 1.73; 95% CI 0.78–3.85; family history: 1.52; 0.82–2.81; age per 10 years: 1.23; 0.99–1.52; *p* = 0.059). Excluding the 62 records with internal contradictions (*n* = 393) strengthened the association with stress (aOR 2.33; 95% CI 1.22–4.48; *p* = 0.011), but family history was no longer significant (1.56; 0.94–2.60; *p* = 0.087). Excluding the 19 outcome-indeterminate records (*n* = 436), or reclassifying them as positive, left family history significant (aOR 1.66 and 1.62) but moved stress to or beyond the threshold (1.83; 0.99–3.41; *p* = 0.055, and 1.68; 0.91–3.13; *p* = 0.100) and made cardiovascular disease significant (2.07; 1.02–4.20, and 2.11; 1.05–4.25). Restricting the analysis to the 417 patients with all four components known (204 events) left all three associations significant (stress 1.97; 1.06–3.66; family history 1.68; 1.04–2.71; cardiovascular disease 2.19; 1.06–4.51). With the mechanical-interdental-cleaning indicator in place of the adjunctive-hygiene-aid indicator, stress remained associated (2.08; 1.13–3.85; *p* = 0.019), family history did not (1.54; 0.96–2.45; *p* = 0.071), and mechanical interdental cleaning was inversely associated with self-reported signs (0.57; 0.37–0.86; *p* = 0.007). Excluding the 87 patients with multiple selections in Q3, Q4 or Q8 (*n* = 368; 163 events) gave a similar estimate for stress (2.33; 1.16–4.70; *p* = 0.018) and a borderline one for family history (1.69; 0.99–2.88; *p* = 0.052). Restriction to patients aged > 20 years, the protocol criterion (*n* = 373; 182 events), strengthened the association with stress (2.87; 1.42–5.82; *p* = 0.003) and with age (1.26 per 10 years; 1.06–1.51), whereas family history was not significant (1.35; 0.80–2.28; *p* = 0.260). Excluding both the September block and the contradictory records (*n* = 216; 114 events; 9.5 events per predictor) left no significant predictor. Using only the five contradiction rules reproducible from item codes, without the dispensary-status flag (which relied on a free-text field not retained in the coded data), left 396 patients and 183 events: stress aOR 2.21 (95% CI 1.16–4.21) and family history 1.53 (95% CI 0.92–2.54) (additional check in Table S8). Across the ten principal specifications the point estimates ranged from 1.68 to 2.87 for stress and from 1.35 to 1.69 for family history, the apparent AUC ranged from 0.664 to 0.693, and the statistical significance of each association depended on the specification. The patient-level associations are therefore not robust and are regarded as exploratory throughout the Discussion.

**Table 8:** Sensitivity analyses of the multivariable model for self-reported periodontal signs: adjusted odds ratios for the two predictors significant in the main model, and model performance.

Analysis	n (Events)	High Stress: aOR (95% CI), *p*	Positive Family History: aOR (95% CI), *p*	Nagelkerke R^2^	AUC (Apparent)
Main model (Table 7)	455 (223)	2.11 (1.14; 3.89), 0.017	1.61 (1.02; 2.55), 0.043	0.123	0.665
(a) Without the 2–4 September 2026 block	251 (140)	1.73 (0.78; 3.85), 0.180	1.52 (0.82; 2.81), 0.182	0.128	0.674
(b) Without 62 records with internal contradictions	393 (181)	2.33 (1.22; 4.48), 0.011	1.56 (0.94; 2.60), 0.087	0.126	0.664
(c) Without 19 outcome-indeterminate records	436 (223)	1.83 (0.99; 3.41), 0.055	1.66 (1.04; 2.65), 0.035	0.140	0.680
(d) 19 outcome-indeterminate records reclassified as positive	455 (242)	1.68 (0.91; 3.13), 0.100	1.62 (1.02; 2.58), 0.042	0.142	0.683
(e) Mechanical interdental aids instead of adjunctive hygiene aids	455 (223)	2.08 (1.13; 3.85), 0.019	1.54 (0.96; 2.45), 0.071	0.137	0.675
(f) All four composite components known	417 (204)	1.97 (1.06; 3.66), 0.032	1.68 (1.04; 2.71), 0.033	0.130	0.668
(g) Without 87 patients with multiple selections in Q3, Q4 or Q8	368 (163)	2.33 (1.16; 4.70), 0.018	1.69 (0.99; 2.88), 0.052	0.127	0.666
(h) Age > 20 years (protocol criterion)	373 (182)	2.87 (1.42; 5.82), 0.003	1.35 (0.80; 2.28), 0.260	0.154	0.693
(a) + (b) combined	216 (114)	1.93 (0.81; 4.60), 0.138	1.65 (0.84; 3.23), 0.147	0.132	0.676

All models contain the 12 predictor terms of Table 7 with simultaneous entry. Analysis (a) was specified after data inspection and analyses (b)–(h) were added during peer review. Outcome-indeterminate records: no observed positive component and at least one unknown component. In analyses (c), (d) and (f), cardiovascular disease became significant (aOR 2.07, 95% CI 1.02–4.20; 2.11, 1.05–4.25; and 2.19, 1.06–4.51); in analysis (h), age became significant (aOR 1.26 per 10 years 95% CI 1.06–1.51); in analysis (e), mechanical interdental cleaning was inversely associated with self-reported signs (aOR 0.57; 95% CI 0.37–0.86; p = 0.007). . *p* values are nominal (unadjusted) and serve exploratory interpretation; the analyses re-estimate one model under alternative assumptions and were not treated as a family of independent tests. aOR, adjusted odds ratio; AUC, area under the receiver operating characteristic curve (apparent, in-sample).

## 4. Discussion

### 4.1. Principal Findings

This study surveys dentists and medical physicians in Kazakhstan about a future periodontal AI tool and includes an exploratory patient survey that did not ask about AI. The findings document declared preferences and barriers; clinical benefits of any proposed function require prospective evaluation of an actual system. Dentists selected history-taking, visual assessment, early gingivitis detection and several radiographic features. These percentages come from separate multi-select questions and do not establish a priority ranking between early clinical tasks and radiographic staging. Autonomy choices were distributed across the five offered clinician-controlled configurations, with low autonomy the most frequent minority choice. The legal framing and absence of an autonomous option limit interpretation. Legal uncertainty was the most commonly cited barrier, but the leading barriers’ confidence intervals overlapped. Experience-related differences did not survive multiplicity adjustment. Physicians and specialists selected early gingivitis screening and patients with diabetes as priorities, but more than half reported insufficient periodontal competence. Absence of significant specialty differences does not establish equivalence. About half of patient responses met the study-specific symptom composite; stress and family-history associations were exploratory and sensitivity-dependent, and diagnostic eligibility, block provenance and unique participation were not verified.

### 4.2. Autonomy, Accountability and Trust in International Perspective

Because every autonomy option in our questionnaire kept the clinician in control, our data describe only the distribution of choices within these five configurations and cannot be compared directly with surveys that measured the acceptance of autonomous AI: the Bulgarian, Australian and Polish surveys explicitly offered respondents a fully automated diagnosis or “AI replacing the dentist” as an option, whereas ours did not, so our data are compatible with those studies but cannot be read as an independent demonstration that dentists in Kazakhstan reject autonomous AI. For context only, in Bulgaria 54.7% of dentists preferred clinician-guided diagnosis and only 3.8% trusted a fully automated diagnosis [32]; in Australia, 91.6% of dentists and students regarded AI as an assistant and named insurance liability and lack of transparency among their principal concerns [31]; in Poland, acceptance of AI replacing the dentist was almost nil (2.7%) despite moderate overall acceptance [34]; and a systematic review of 13 studies concluded that dentists consistently frame AI as a supplementary tool [30]. The Romanian multi-stakeholder survey provides the closest parallel to our liability finding: 74.0% of respondents would refrain from using AI while legal liability remained unclear, 87.8% would not trust an unverifiable recommendation, and responsibility was attributed primarily to clinicians (77.4%) [39]. Most of our dentists who answered the open question on responsibility expressed a similar view (for example, “responsibility lies entirely with the doctor”), while a few described shared or split responsibility, in one case extending to the developer for system failures (Section 3.2; descriptive tally, not a formal qualitative analysis)—an allocation that legal scholarship has long identified as unresolved [29]. What differs is the ranking of barriers. In Kuwait, lack of training (71.2%), technical challenges (63.4%) and cost (56.5%) dominated [33], and in Egypt educational gaps were the main determinant of adoption among periodontists [36], whereas in our sample the absence of a legal framework was cited most often and cost and equipment least often, although the leading barriers could not be ranked reliably. A plausible explanation is timing: our survey was conducted within a year of the adoption of Kazakhstan’s Law “On Artificial Intelligence”, which introduced risk-based classification, transparency and user-notification obligations [41,42]; we did not analyse whether sectoral health-care rules on clinical liability or on the approval of AI as a medical device have since been adopted, and our data show only that respondents perceived the legal framework as insufficient. The salience of regulation among our respondents may therefore reflect perceived regulatory uncertainty rather than a cultural peculiarity, much as the risk-based obligations of the European Union AI Act [43] and the WHO governance principles [44] have shaped the discourse elsewhere.

The experience gradient we observed at the nominal level—less experienced dentists more often choosing “low autonomy” defined as AI processing data only, more experienced dentists more often choosing explicit verification rules and formal prohibition of full autonomy—has not been reported in this form before, although age and experience have been associated with attitudes in several surveys, in inconsistent directions [30,34]. One speculative interpretation, consistent with the framework of autonomy levels proposed by Bitterman et al. [25], is that experienced clinicians reason about AI in terms of accountability structures (who verifies, who is liable), whereas less experienced clinicians reason in terms of the technical scope of the tool. Younger dentists were also more likely to distrust automated output (73.3% vs. 33.3%), which contrasts with the higher AI use reported among younger and more highly educated dental professionals elsewhere [35]. These subgroup results rest on 15 and 18 respondents, did not survive adjustment for multiple comparisons, and must be replicated before any interpretation is attached to them.

### 4.3. The Role of Primary Care

The physicians’ responses are compatible with the international pattern of high awareness but limited practice, although our survey measured neither their knowledge of the periodontal–systemic evidence nor their actual screening or referral practice. In Switzerland, all surveyed endocrinologists and general practitioners recognised periodontitis as inflammatory and 82.6% linked poor glycaemic control to severe periodontal disease, yet 34.8% and 20.4% respectively never or rarely referred patients to a dentist, citing insufficient training and time [12]; Irish primary care physicians rarely implemented oral-health recommendations for diabetes [13]; and a scoping review of the integration of oral health into primary care identified competence, time, reimbursement and the lack of simple tools as recurrent barriers [11]. Our respondents named exactly these barriers—competence (53.8%), patient motivation (51.6%), time (42.9%) and the absence of a simple instrument (41.8%)—while simultaneously endorsing early gingivitis screening as their most effective contribution (80.2%). This combination suggests a hypothesis rather than a product that the physicians themselves requested: that a low-threshold screening tool for the PHC consultation that does not require periodontal expertise could help, for example automated analysis of intraoral photographs—in a development study, gingival inflammation was detected on standardised intraoral photographs taken with a digital single-lens reflex camera, with pixel-level sensitivity of 0.92 and specificity of 0.94 in a held-out validation set [16], a pilot external validation among older adults in community daycare centres found lower specificity [17], and smartphone-based screening in routine primary care would require separate validation—or a short validated self-report instrument such as the rapid periodontitis screening tool developed for medical settings [52]. Such a tool would operationalise the EFP/WONCA recommendation that family physicians screen and refer [6] and the WHO action-plan target of integrating oral health into PHC [7]. The recommendation remains speculative, however: the survey assessed neither physicians’ actual screening behaviour or performance nor the time, workflow and reimbursement conditions of the consultation, so whether such a tool is feasible in routine PHC practice in Kazakhstan, and whether physicians would use it, are questions for feasibility and implementation studies. Both groups most often selected early gingivitis as the priority stage, and diabetes was among the comorbidities most often selected by both, but this does not establish agreement between the professions (Section 3.4), and the survey did not ask whether a shared data model would be acceptable. Some dentists’ open-ended answers nevertheless described such a model, for example “one database” or a dashboard on which “all doctors see the overall course of treatment” (illustrative quotations only). A shared record would, however, raise questions of data privacy and patient consent, interoperability between dental and medical information systems, differentiated access permissions and information security that this survey did not evaluate and that would have to be resolved under Kazakhstan’s personal-data and health-care legislation before any such record is designed.

### 4.4. Patient-Level Evidence for Algorithm Inputs

Clinicians proposed diabetes, oral hygiene and smoking as the principal inputs for a periodontal algorithm. The exploratory patient data can only generate hypotheses about these inputs, because the outcome was self-reported signs and diagnostic eligibility was unverified. The univariable association between dysglycaemia and self-reported signs (OR 3.52) is consistent with the established bidirectional relationship [3,5,53], and the association with cardiovascular disease (OR 2.83) with the EFP/World Heart Federation consensus [4]; the attenuation of both after adjustment for age is expected given the small number of affected patients (32 with dysglycaemia, 64 with cardiovascular disease) and the correlation of both conditions with age. The adjusted association with high stress (OR 2.11) is in the direction of a cross-sectional study reporting an association between psychological stress and periodontitis [54] and of the psychobiological pathways described for stress-related diseases and periodontal wound healing [55], and the association with a positive family history (OR 1.61) is in line with a systematic review estimating that up to a third of the population variance in periodontitis is attributable to genetic factors [56]. Both associations, however, were modest, close to the significance threshold, and lost significance in several sensitivity analyses (Table 8); their clinical relevance should not be overstated. By contrast, current smoking was not associated with the composite indicator or with bleeding, despite the well-established effect of smoking on periodontitis (pooled relative risk about 1.85) [57]. A possible explanation, which we could not test directly, lies in the composition of the outcome: smoking is known to suppress gingival bleeding through vasoconstriction, so a self-reported indicator that includes bleeding may be less sensitive in smokers (bleeding contributed to 92 of the 223 positive classifications and gingival appearance to 138). Consistent with this hypothesis, smoking was associated with halitosis, suppuration and xerostomia but not with bleeding in our data, but the explanation remains untested. Similarly, toothbrushing frequency and dental attendance were unrelated to self-reported signs; brushing correlated with attendance but not with bleeding, which may indicate that in this sample hygiene behaviour indexes health consciousness more than plaque control, although mechanical interdental cleaning was inversely associated with signs in a sensitivity analysis added during revision. For algorithm design, these results suggest that stress and family history—variables that clinicians did not rank highly (stress was proposed by 42.4% of dentists and 34.1% of physicians, heredity by 44.0% of physicians)—are candidate inputs worth examining, and they raise the question whether self-reported bleeding should be complemented by other symptoms in smokers. Whether these variables add predictive value must be established on clinically validated periodontal outcomes in independent datasets before they are built into any algorithm; the present data do not justify their inclusion.

The low internal consistency of the six symptom items (α = 0.56) is itself informative. In a large United States sample, models combining the CDC/American Academy of Periodontology self-report questions—a different set from ours—with demographic variables predicted clinically defined periodontal outcomes (periodontitis and clinical attachment loss) with AUCs of about 0.8 [48], and validated rapid screening instruments exist for medical settings [52]. Our ad hoc items, which mix frequency, severity and appearance across different response formats, do not reach this standard; our composite indicator shares some content with these questions but is not the same instrument, uses its own thresholds, has not been validated against clinical examination, and should be regarded as a pragmatic outcome rather than a validated measure of periodontal disease. An AI system that relies on patient-reported data will need locally validated instruments in Russian and Kazakh.

### 4.5. Implications for the Design and Governance of Periodontal AI

Table 9 summarises the findings as proposed requirements for a future system. Each entry is classified by the type of evidence behind it: (A) a preference directly measured by the survey; (B) an exploratory association in the patient data that requires confirmation; or (C) a recommendation that rests on external evidence or on our interpretation rather than on the survey. None of the entries has been prioritised formally or assessed for technical feasibility, cost or clinical benefit, so the table is a starting point for requirement validation with users (for example by Delphi or usability methods), not a specification. Within their respective questions, the options most often selected by clinicians concerned early detection of gingivitis, structured history-taking, photograph-based assessment and remote monitoring, with a graphical periodontal chart as the most frequently chosen output and an ICD-coded text summary next. Respondents selected several systemic and behavioural variables as possible inputs, but the architecture connecting them is an author-derived proposal; interprofessional access is supported only by illustrative open-ended answers, and stress and family history are candidate inputs only. In terms of human–AI interaction, all five configurations offered—and therefore all choices—correspond to the lower autonomy levels described by Bitterman et al. [25], so the survey cannot indicate an acceptable upper limit of autonomy; within this range choices were spread across the options. The interface might expose source data and the allocation of responsibility, the two most frequent overall transparency choices (36.4% and 30.3% respectively), in keeping with the transparency and explainability requirements of the ethical frameworks for dental AI [23,24,26]; whether an autonomous mode would be rejected was not tested. Governance-wise, the absence of a legal framework and liability insurance was the barrier most often cited by dentists, which identifies clarification of liability under the national AI law and the health-care legislation as a perceived need (the survey did not ask whether it is a precondition for adoption), echoing the observation that trust in health-care AI depends on institutional as much as on technical assurances [27,28]. This perceived need corresponds to the final stage—legal governance covering liability and insurance—of the phased implementation model proposed for periodontal AI in Kazakhstan [20].

### 4.6. Strengths and Limitations

The strengths of the study are the simultaneous survey of dental and medical clinicians within a single framework, complemented by an exploratory patient survey, the comparatively large patient sample, the transparent documentation of data processing (Figure 1), the post-collection form printouts, codebooks and analysis code (Supplementary Materials S2–S5), the systematic use of confidence intervals and effect sizes, the explicit accounting for multiple exploratory comparisons, and the sensitivity analyses. Several limitations must be acknowledged. The cross-sectional design precludes causal inference. The most important limitation of the clinician findings is the design of the autonomy item: all five response options preserved clinician control and no option described an autonomous system, so the principal finding that dentists chose only clinician-controlled configurations is a property of the questionnaire, not an empirical demonstration of opposition to autonomous AI; the options also mixed technical scope, verification arrangements and a normative position, while the legal framing mixed perceived permissibility with personal attitudes. The questionnaires have preliminary face and content validity only: expert review and a small pilot do not establish measurement validity, the expert review was internal to the working group, the Kazakh version was a single forward translation without back-translation, language equivalence and the timing of bilingual wording could not be established from the retained documentation, comprehension was not compared between language groups, and no validated scales of AI knowledge, experience or readiness were included.

Periodontal status was self-reported without clinical indices; the patient information sheet specified K05.4 and age > 20 years, but neither the actual diagnostic mix nor chart findings were available for this analysis, treatment status was not clinically verified, more than half of the patients denied ever having had symptoms, and the composite indicator is a pragmatic study-specific outcome, not a validated measure of periodontal disease, so the 49.0% figure describes symptom reporting in the achieved sample and not disease prevalence.

Sampling was non-probabilistic: dentists were few, mostly private practitioners from Astana, endocrinologists, gastroenterologists and haematologists were few among physicians, recruitment denominators and response rates are unknown, and the findings are not generalisable to clinicians or patients in Kazakhstan. The inclusion criteria were not fully enforced (15 dentists had ≤5 years of experience and 82 patients were aged 18–20 years). The anonymous online forms could not technically prevent repeat submission, and eligibility was self-declared; near-duplicate screening cannot verify identity or unique participation. Historical consent-item validation and individual consent selections were not retained in the coded files. The required status of open questions in the later printouts is inconsistent with the retained answer counts, and the documentation does not explain whether settings changed or answers were removed during preparation. The extensively bilingual later printouts also differ from the authors’ description of predominantly Russian export labels, so equivalence to every collection-period form state cannot be assumed.

A block of 204 patient responses received within three days had a highly repetitive age structure, closely spaced submission timestamps, which are not individual completion times, and a different risk profile; it coincided with dissemination through an inter-university platform, so many of its respondents may not have been patients invited by clinicians, its individual-level eligibility could not be established, and its exclusion removed the statistical significance of all predictors; the patient-level associations are therefore not robust and are preliminary. Five items intended as single-choice were implemented as checkboxes and received multiple selections, and comparison with the approved paper versions (S6) identifies three option-set differences: two dentist options absent from the later printouts and one physician option added online; the timing of these changes relative to the start of data collection could not be independently verified (Supplementary Material S5; Table S6). These response-type and option-set problems affect the interpretation of the physicians’ ordinal item, the dentists’ re-assessment item and the patients’ mobility, gingival-appearance and hygiene items; restriction to single answers changed the distributions of the two clinician items but not the direction of the principal patient associations. The original hygiene indicator grouped mouthwash with mechanical aids, which was examined in a sensitivity analysis, and the classification of 19 outcome-indeterminate patients rested on the assumption that unknown components were absent. Sixty-two patient records contained internal contradictions that were resolved in favour of the reported sign. One flag affecting five records relied on a free-text diagnosis field that was not retained in the coded data and therefore cannot be re-derived; an analysis using only documented rules is shown in Table S8.

The small dentist sample (*n* = 33) limits the power of all clinician comparisons, the associations with professional experience rest on subgroups of 15 and 18 respondents and did not survive adjustment for multiple testing, and the dentist–physician comparisons rely on items with largely identical option labels but different stems for comorbidities, and only partly comparable wording for clinical stage. Finally, most effect sizes among patients were small (φ ≤ 0.18; r ≤ 0.30; |ρ| ≤ 0.37), the multivariable model explained only about 12% of the variance with an apparent AUC of 0.665 that was not validated, and the identified factors are candidate inputs rather than a predictive model. A comparable limitation was observed in a five-year analysis of emergency medical service data from Astana, in which routinely coded cardiac comorbidities were independently associated with the prehospital transport decision, but the model achieved only moderate discrimination (AUC 0.69) and was not recommended for individual triage without prospective validation and additional clinical data [58].

### 4.7. Future Research

Two validation steps should precede any prototype development. First, the three questionnaires need independent psychometric validation in Russian and Kazakh, including an autonomy item that offers a genuinely autonomous option, validated scales of AI readiness, and testing of comprehension in both language groups. Second, the patient-reported outcomes need clinical verification: the self-report items should be validated against periodontal examination (probing depth, clinical attachment level, bleeding on probing) in Russian- and Kazakh-speaking patients, and the candidate associations with stress and family history should be re-examined on clinically defined outcomes in an independent sample. Only then should a prototype decision-support tool for the PHC setting be developed and evaluated for its accuracy against clinical periodontal examination, its acceptability under different autonomy configurations, its feasibility within the consultation and its effect on referral behaviour. A probability-based survey of dentists across the regions of Kazakhstan and comparative analyses with neighbouring Central Asian countries would strengthen the evidence base, and qualitative work with regulators, insurers and developers is needed to translate the demand for liability rules into concrete provisions under the national AI law.

## 5. Conclusions

The clinician surveys identified preliminary preferences for a future periodontal AI system, including early gingivitis detection, structured data collection and graphical outputs, alongside perceived barriers involving competence, usability and accountability. The autonomy question describes choices among five clinician-controlled configurations under a legal-permissibility framing; it establishes neither rejection of autonomous AI nor a validated preference hierarchy. A simple PHC screening mode and shared record remain proposals for feasibility and user validation, rather than evaluated interventions. The patient questionnaire did not measure attitudes towards AI. Its self-reported signs and associations with stress and family history are exploratory, sensitive to data handling and sample composition, and do not describe disease prevalence or a clinically verified diagnostic cohort. The 204-response block, collected after dissemination through an inter-university platform, may include respondents other than clinically invited patients; its individual eligibility and unique participation remain unverified. The study evaluates no AI system and establishes neither a validated design framework nor clinical readiness. Independent instrument validation, clinical outcome verification, clarification of recruitment and consent documentation, and feasibility assessment should precede use of these findings in system design or implementation.

## Figures and Tables

**Table 1:** Characteristics of the study participants.

Characteristic	Dentists (*n* = 33)	PHC Physicians and Specialists (*n* = 91)	Patients (*n* = 455)
Age, years: Me (Q1–Q3)	33 (24–40)	42 (35–55.8) ^1^	40 (22–47)
Age, years: min–max	23–53	22–70	18–77
Men, *n* (%)	21 (63.6)	12 (13.2)	148 (32.5)
Women, *n* (%)	12 (36.4)	79 (86.8)	307 (67.5)
Residence/work in Astana, *n* (%)	28 (84.8)	28 (30.8)	288 (63.3)
Polyclinic, *n* (%)	2 (6.1)	66 (72.5)	–
Hospital, *n* (%)	2 (6.1)	11 (12.1)	–
Private clinic, *n* (%)	29 (87.9)	13 (14.3)	–
Professional experience < 1 year, *n* (%)	3 (9.1)	5 (5.5)	–
Professional experience 1–5 years, *n* (%)	12 (36.4)	9 (9.9)	–
Professional experience 5–10 years, *n* (%)	3 (9.1)	18 (19.8)	–
Professional experience 10–15 years, *n* (%)	5 (15.2)	19 (20.9)	–
Professional experience > 15 years, *n* (%)	10 (30.3)	40 (44.0)	–
Age 18–29 years, *n* (%)	–	–	152 (33.4)
Age 30–44 years, *n* (%)	–	–	181 (39.8)
Age 45–59 years, *n* (%)	–	–	74 (16.3)
Age ≥ 60 years, *n* (%)	–	–	48 (10.5)
On dispensary register, *n* (%)	–	–	42 (9.2)

^1^ *n* = 90 (one implausible value set to missing). Me, median; Q1, Q3, first and third quartiles; PHC, primary health care.

**Table 2:** Most frequent responses of dentists (*n* = 33) to the closed items on the requirements for an AI-enabled periodontal decision-support system.

Item	Three Most Frequent Options: *n* (%) [95% CI, %]
Q1. Examination procedures to automate first	History-taking and complaints, 27 (81.8) [65.6–91.4]; visual assessment of the gingiva, 24 (72.7) [55.8–84.9]; probing of pocket depth, 21 (63.6) [46.6–77.8]
Q2. Radiographic signs on panoramic radiographs	Destruction of the cortical plate of the interdental septa, 30 (90.9) [76.4–96.9]; widening of the periodontal ligament space, 25 (75.8) [59.0–87.2]; depth of bony pockets, 22 (66.7) [49.6–80.2]
Q3. Comorbid conditions for the AI algorithm	Diabetes mellitus, 27 (81.8) [65.6–91.4]; osteoporosis, 22 (66.7) [49.6–80.2]; hormonal disorders, 21 (63.6) [46.6–77.8]
Q4. Indices difficult to interpret without digital assistance	PMA index, 20 (60.6) [43.7–75.3]; Greene–Vermillion index, 19 (57.6) [40.8–72.8]; CPITN, 16 (48.5) [32.5–64.8]
Q5. Risk factors for the prevention plan	Quality of oral hygiene, 28 (84.8) [69.1–93.3]; smoking duration, 24 (72.7) [55.8–84.9]; malocclusion, 24 (72.7) [55.8–84.9]
Q6. Stage at which AI is most needed	Early diagnosis of gingivitis, 23 (69.7) [52.7–82.6]; differential diagnosis of mild vs. moderate periodontitis, 20 (60.6) [43.7–75.3]; screening of healthy individuals, 19 (57.6) [40.8–72.8]
Q7. Configuration considered permissible under Kazakhstan legislation (single choice; all five options shown)	Low autonomy (AI processes data only), 10 (30.3) [17.4–47.3]; medium autonomy, 6 (18.2) [8.6–34.4]; decision support with mandatory verification, 6 (18.2) [8.6–34.4]; AI as a “second opinion”, 6 (18.2) [8.6–34.4]; full autonomy prohibited, 5 (15.2) [6.7–30.9]. All five options preserved clinician control
Q8. Laboratory data to be matched	Glucose > 6 mmol/L, 23 (69.7) [52.7–82.6]; leukocytes, 22 (66.7) [49.6–80.2]; cytokines (IL-8, IL-2), 22 (66.7) [49.6–80.2]
Q9. Priority AI function	Remote monitoring, 22 (66.7) [49.6–80.2]; index calculation from photographs, 20 (60.6) [43.7–75.3]; predictive analysis of tooth loss, 19 (57.6) [40.8–72.8]
Q10. Anatomical risk factors	Tooth crowding, 24 (72.7) [55.8–84.9]; mucosal cords, 21 (63.6) [46.6–77.8]; abnormal frenula, 21 (63.6) [46.6–77.8]
Q11. Frequency of risk re-assessment	Four times a year with diabetes/CVD, 24 (72.7) [55.8–84.9]; twice a year without somatic disease, 18 (54.5) [38.0–70.2]; monthly through an app, 14 (42.4) [27.2–59.2]
Q12. Preferred output format	Graphical periodontal chart, 22 (66.7) [49.6–80.2]; text with ICD-10 code, 19 (57.6) [40.8–72.8]; probabilistic prognosis (%), 18 (54.5) [38.0–70.2]
Q13. Most important aspect of transparency (single choice)	Access to source data, 12 (36.4) [22.2–53.4]; delimitation of responsibility, 10 (30.3) [17.4–47.3]; explanation of the reasoning, 7 (21.2) [10.7–37.8]
Q14. Criteria of treatment success	Reduction in probing depth, 25 (75.8) [59.0–87.2]; stabilisation of septal height, 23 (69.7) [52.7–82.6]; reduced mobility, 19 (57.6) [40.8–72.8]
Q15. Barriers to AI integration	No legal framework/liability insurance, 20 (60.6) [43.7–75.3]; complex interface and IT skills, 18 (54.5) [38.0–70.2]; workload and lack of time, 17 (51.5) [35.2–67.5]

Percentages are calculated from the number of respondents and may sum to more than 100% within an item. Q11 had no multi-select wording in the approved paper version (S6) but displayed the standard checkbox instruction in the later form; its frequencies referred to different comorbidity or delivery settings: 20 of 33 respondents selected more than one frequency, and among the 13 who selected a single option, twice a year was chosen by 6, four times a year by 5 and once a year by 2 (Tables S6 and S11). CI, Wilson confidence interval; PMA, papillary–marginal–alveolar index; CPITN, Community Periodontal Index of Treatment Needs.

**Table 3:** Responses to the legally framed autonomy item and priority aspect of transparency according to dentists’ professional experience, *n* (%).

Response Option	≤5 Years (*n* = 15)	>5 Years (*n* = 18)
Q7. Low autonomy (AI processes data only)	7 (46.7)	3 (16.7)
Q7. Medium autonomy	3 (20.0)	3 (16.7)
Q7. Decision support with mandatory verification	1 (6.7)	5 (27.8)
Q7. AI as a “second opinion”	4 (26.7)	2 (11.1)
Q7. Full autonomy prohibited	0 (0.0)	5 (27.8)
Q7: χ^2^(4) = 9.74, p = 0.045; exact p = 0.045; Cramér’s V = 0.54		
Q13. Access to source data	8 (53.3)	4 (22.2)
Q13. Explanation of the reasoning	4 (26.7)	3 (16.7)
Q13. Operational limitations	2 (13.3)	0 (0.0)
Q13. Labelling of synthetic results	0 (0.0)	2 (11.1)
Q13. Delimitation of responsibility	1 (6.7)	9 (50.0)
Q13: χ^2^(4) = 11.70, p = 0.020; exact p = 0.012; Cramér’s V = 0.60		

Pearson’s χ^2^ and Fisher–Freeman–Halton exact test for 2 × 5 tables. Expected frequencies were below 5 in 9 of 10 cells for Q7 (minimum 2.27) and 7 of 10 cells for Q13 (minimum 0.91); inference is based on the exact *p* value. Neither association remained significant after Holm–Bonferroni adjustment across the 71 experience comparisons; both are exploratory.

**Table 4:** Most frequent responses of PHC physicians and medical specialists (*n* = 91) to the items not shown in Figure 3.

Item	Three Most Frequent Options: *n* (%) [95% CI, %]
Q4. Importance of periodontal status for diabetes compensation	Important (indicator of angiopathy), 46 (50.5) [40.5–60.6]; very important, 45 (49.5) [39.4–59.5]; extremely critical, 42 (46.2) [36.3–56.3]
Q5. Anamnestic factors of severe periodontitis	Duration of the systemic disease, 69 (75.8) [66.1–83.5]; smoking duration, 61 (67.0) [56.9–75.8]; heredity, 40 (44.0) [34.2–54.2]
Q6. Comorbidities for automatic AI alerts	Gastrointestinal diseases, 61 (67.0) [56.9–75.8]; diabetes mellitus, 57 (62.6) [52.4–71.9]; osteoporosis, 47 (51.6) [41.5–61.6]
Q7. Complete blood count findings warranting referral to a periodontist	Leukocytosis/leukopenia, 53 (58.2) [48.0–67.8]; raised ESR without a focus, 52 (57.1) [46.9–66.8]; aplastic anaemia/agranulocytosis, 47 (51.6) [41.5–61.6]
Q8. Complaints indicating gingivitis	Gingival bleeding, 70 (76.9) [67.3–84.4]; periodic swelling, 58 (63.7) [53.5–72.9]; halitosis, 53 (58.2) [48.0–67.8]
Q10. Best indicator of quality-of-life impact (single choice)	Emergency visits, 30 (33.0) [24.2–43.1]; economic costs, 22 (24.2) [16.5–33.9]; restrictions in food choice, 19 (20.9) [13.8–30.3]
Q12. Periodontal changes in gastrointestinal disease	Catarrhal/ulcerative processes, 64 (70.3) [60.3–78.7]; persistent plaque, 50 (54.9) [44.7–64.8]; recurrent course, 49 (53.8) [43.7–63.7]

Percentages are calculated from the number of respondents and may sum to more than 100% within an item. Barriers to oral examination (Q9), the stage of PHC involvement (Q11) and priority groups for AI-assisted screening (Q13) are shown with all response options and 95% CIs in Figure 3 and are not repeated here. Q4 was designed as a single ordinal rating but allowed multiple selections online (34 of 91 respondents selected more than one level; Table S6), so its percentages overlap; among the 57 single answers, “extremely critical” was chosen by 16 (28.1%) (Table S11). CI, Wilson confidence interval; ESR, erythrocyte sedimentation rate; PHC, primary health care.

**Table 5:** Comparison of dentists and PHC physicians/specialists on overlapping questionnaire items.

Item	Dentists (*n* = 33), *n* (%)	Physicians (*n* = 91), *n* (%)	OR (95% CI)	*p* (Holm *p*)	φ
Diabetes mellitus	27 (81.8)	57 (62.6)	2.68 (1.01; 7.16)	0.052 (0.412)	0.18
Gastrointestinal diseases	18 (54.5)	61 (67.0)	0.59 (0.26; 1.33)	0.212 (1.000)	0.11
Cardiovascular diseases	16 (48.5)	39 (42.9)	1.25 (0.56; 2.79)	0.683 (1.000)	0.05
Respiratory diseases	11 (33.3)	32 (35.2)	0.92 (0.40; 2.14)	1.000 (1.000)	0.02
Hormonal disorders	21 (63.6)	41 (45.1)	2.13 (0.94; 4.85)	0.103 (0.723)	0.16
Osteoporosis	22 (66.7)	47 (51.6)	1.87 (0.81; 4.30)	0.156 (0.938)	0.13
Immunodeficiency states	19 (57.6)	47 (51.6)	1.27 (0.57; 2.84)	0.684 (1.000)	0.05
Stage: early diagnosis/screening of gingivitis	23 (69.7)	73 (80.2)	0.57 (0.23; 1.40)	0.231 (1.000)	0.11
Stage: monitoring of remission	18 (54.5)	25 (27.5)	3.17 (1.39; 7.23)	0.010 (0.087)	0.25

Fisher’s exact test (two-sided); Holm-adjusted *p* across the nine comparisons in parentheses. OR, odds of selection by dentists relative to physicians, with Woolf 95% CI. The first seven rows refer to comorbid conditions to be considered by the AI algorithm.

**Table 6:** Frequency of self-reported signs of periodontal disease according to behavioural and systemic factors (patients, *n* = 455).

Factor	Factor Present: n/N (%)	Factor Absent: n/N (%)	OR (95% CI)	χ^2^(1)	*p*	φ
Female sex	159/307 (51.8)	64/148 (43.2)	1.41 (0.95; 2.09)	2.92	0.087	0.08
Current smoking	30/58 (51.7)	193/397 (48.6)	1.13 (0.65; 1.97)	0.20	0.658	0.02
Toothbrushing < 2 times/day	71/137 (51.8)	152/318 (47.8)	1.17 (0.79; 1.75)	0.62	0.431	0.04
High stress level	43/62 (69.4)	180/393 (45.8)	2.68 (1.51; 4.76)	11.89	<0.001	0.16
Dysglycaemia ^1^	24/32 (75.0)	183/398 (46.0)	3.52 (1.55; 8.04)	9.99	0.002	0.15
Cardiovascular disease	45/64 (70.3)	178/391 (45.5)	2.83 (1.60; 5.02)	13.52	<0.001	0.17
Positive family history ^1^	79/129 (61.2)	112/266 (42.1)	2.17 (1.41; 3.34)	12.74	<0.001	0.18
Dental visits ≥ once a year	117/244 (48.0)	106/211 (50.2)	0.91 (0.63; 1.32)	0.24	0.627	0.02

Pearson’s χ^2^ test (all expected frequencies ≥ 5); OR with Woolf 95% CI. Holm-adjusted *p* across the eight comparisons: 0.003 (stress), 0.008 (dysglycaemia), 0.002 (cardiovascular disease) and 0.003 (family history); results without the 2–4 September 2026 block are given in Table S10. ^1^ Patients who had never had their glucose measured (*n* = 25) or who did not know their family history (*n* = 60) were excluded from the respective comparison.

**Table 9:** Proposed requirements for an AI-enabled periodontal decision-support system derived from the two clinician surveys and the exploratory patient analysis, classified by the type of supporting evidence.

Domain	Proposed Requirement	Evidence Type and Supporting Data From This Study
Clinical scope	Support early gingivitis detection, screening of at-risk individuals and remission monitoring; development order requires validation	A. Measured response frequencies: early gingivitis 69.7% of dentists and 80.2% of physicians; remission monitoring 54.5% vs. 27.5% (nominal *p* = 0.010; Holm *p* = 0.087). C. Any development hierarchy is an author-derived proposal; no cross-domain prioritisation was measured
Input data	Structured history-taking; photograph-based gingival assessment; radiographic markers (cortical plate, periodontal ligament space, bony pockets); glycaemic status, cardiovascular disease, smoking, hygiene as clinician-proposed inputs; stress and family history as candidate inputs to be tested	A. Directly measured preference: automation of history-taking (81.8%) and visual assessment (72.7%); diabetes proposed by 81.8% of dentists. B. Exploratory association: stress and family history associated with self-reported signs (aOR 2.11 and 1.61), not robust in sensitivity analyses; requires confirmation on clinically verified outcomes C. Integration of these inputs and the model architecture are author-derived proposals, not measured requirements.
Target groups	Clinician-proposed groups: patients with diabetes, adults ≥65 years, smokers and workers in hazardous industries; eligibility for a future tool requires separate validation	A. Directly measured preference: physicians’ priority groups diabetes 71.4%, ≥65 years 62.6%, hazardous industries 52.7%, smokers 50.5%
Output	Graphical periodontal chart with colour-coded risk zones; ICD-coded text summary; probabilistic prognosis as optional layer; frequency of risk re-assessment to be determined	A. Chart 66.7%, ICD-10 text 57.6%, probability 54.5%. Re-assessment choices referred to different comorbidity or delivery settings and allowed multiple selections; they do not identify one frequency. C. Output integration and re-assessment policy require validation
Human–AI interaction	Evaluate clinician-controlled configurations in future user studies; acceptable upper autonomy limit is unknown	A. Distribution across five offered configurations (low autonomy 30.3%; others 15.2–18.2%; no majority) under a legal-permissibility framing. C. A deployment rule or exclusion of autonomous AI would depend on external evidence and regulation, not this question
Transparency	Expose source data to the user; explain the reasoning; display the allocation of responsibility for each recommendation	A. Overall transparency choices: source data 36.4%, allocation of responsibility 30.3%, explanation 21.2%. Experience comparisons are exploratory. C. Requiring all three interface features is an author proposal informed by ethical frameworks [23,24,26]
Interprofessional use	Shared patient record and dashboard accessible to dentists and PHC physicians; simple screening mode for physicians without periodontal training; data privacy, consent, interoperability, access permissions and information security to be specified	A. Directly measured barriers: physicians’ insufficient competence 53.8%, no simple tool 41.8%. C. A simple screening mode and a shared record are the authors’ inferences from these barriers and from illustrative open-ended answers (e.g., “one database”; no formal qualitative analysis); privacy, interoperability, access and security requirements were not evaluated in this study and derive from the authors’ interpretation and legislation
Governance	Clarify liability and insurance under the national AI law and health-care legislation before deployment; provide training and a usable interface	A. Directly measured barriers: no legal framework/insurance 60.6%; interface complexity 54.5%; distrust of output 51.5% (whether these are preconditions for adoption was not asked). C. The specific legal provisions needed derive from the authors’ reading of the AI law [41,42] and the implementation model [20]
Measurement	Use locally validated self-report instruments; examine whether bleeding items should be complemented by other symptoms in smokers	B. Derived from the patient data: six ad hoc symptom items not reliable (α = 0.56); smoking unrelated to bleeding but related to halitosis, suppuration and xerostomia (explanation untested). C. Use of validated instruments rests on external evidence [48,52]

Evidence types: A, preference directly measured by the survey; B, exploratory association in the patient data requiring confirmation; C, recommendation based on external evidence or the authors’ interpretation. aOR, adjusted odds ratio; PHC, primary health care.

## Data Availability

The codebooks, the analysis code and the output of the archived analysis run are provided in Supplementary Materials S3 and S4; the code runs on the anonymised coded datasets. The anonymised datasets supporting the findings of this study are available from the corresponding author on reasonable request.

## Supplementary Materials

The following are available online at https://www.mdpi.com/article/10.3390/healthcare14203554/s1, Supplementary Material S1: CROSS checklist (Consensus-Based Checklist for Reporting of Survey Studies); Supplementary Material S2: Questionnaire coding and English translation; Supplementary Material S3: Codebooks and verification report (Supplementary_S3_Codebooks_and_verification.xlsx); Supplementary Material S4: Analysis code (Supplementary_S4_analysis_code.py) and the output of its archived run (Supplementary_S4_output.xlsx); Supplementary Material S5: Post-collection questionnaire documentation (Supplementary_S5_Questionnaire_documentation.pdf); Supplementary Material S6: Participant-information materials and the approved paper questionnaires with consent forms (Supplementary_S6_Participant_information.pdf); Table S1: Response options of dentists whose frequency differed by professional experience at the nominal level (unadjusted p < 0.05; 7 of 69 comparisons), with Holm-adjusted p values; Table S2: Descriptive statistics of the ordinal patient items (higher score = less favourable state); Table S3: Item characteristics of the candidate six-item symptom scale (patients with complete answers, *n* = 399); Table S4: Severity of individual symptoms according to behavioural and systemic factors: effect size r (p), Mann–Whitney U test, with Holm–Bonferroni adjustment across the 56 comparisons; Table S5: Severity of symptoms according to age group and smoking status (Kruskal–Wallis test), with Holm-adjusted p values; Table S6: Questionnaire implementation discrepancies and the scope of historical version verification; Table S7: Principal findings of the physician survey by specialty group, *n* (%); Fisher–Freeman–Halton exact test across the four groups and Fisher's exact test for PHC physicians (general practitioners, internists and paediatricians; *n* = 62) versus medical specialists (*n* = 29), with Holm-adjusted p values in parentheses; Table S8: Sensitivity analyses of the multivariable logistic regression for self-reported periodontal signs: adjusted odds ratios (95% CI) for all predictors; Table S9: Spearman rank correlations between symptoms, age and behavioural factors in patients (pairwise *n* = 398–455), with Holm adjustment; Table S10: Univariable associations with self-reported periodontal signs without the 2–4 September 2026 response block (*n* = 251; 140 with signs), compared with the full sample; Table S11: Items designed as single-choice but implemented with multiple selection online: distribution among all respondents and among respondents who selected a single option; Table S12: Dentists' open-ended answers on responsibility following discordant AI and clinician diagnoses and a patient complication (Q20): descriptive tally (29 of 33 dentists answered); Table S13: Characteristics of the 2–4 September 2026 response block and the remaining patient sample; Figure S1: Comorbid conditions to be taken into account by an AI algorithm (dentists) or to trigger an automatic AI alert (physicians): comparison of dentists (*n* = 33) and PHC physicians/specialists (*n* = 91).

## Abbreviations

The following abbreviations are used in this manuscript:

AI	Artificial intelligence
aOR	Adjusted odds ratio
AUC	Area under the receiver operating characteristic curve
CDSS	Clinical decision-support system
CHERRIES	Checklist for Reporting Results of Internet E-Surveys
CI	Confidence interval
CPITN	Community Periodontal Index of Treatment Needs
CROSS	Consensus-Based Checklist for Reporting of Survey Studies
EFP	European Federation of Periodontology
ESR	Erythrocyte sedimentation rate
ICD-10	International Classification of Diseases, 10th Revision
IL	Interleukin
Me	Median
OR	Odds ratio
PHC	Primary health care
PMA	Papillary–marginal–alveolar index
Q1, Q3	First and third quartiles
SE	Standard error
STROBE	Strengthening the Reporting of Observational Studies in Epidemiology
VIF	Variance inflation factor
WHO	World Health Organization
WONCA	World Organization of Family Doctors
