# Supplementary material for: Stakeholder-Reported Requirements for Future Artificial Intelligence Decision Support in Periodontal Care: A Cross-Sectional Survey of Dentists, Physicians and Patients in Kazakhstan
Source: Healthcare (Basel). 2026 Oct 21;14(20):3554. doi: 10.3390/healthcare14203554 (PMC13704399; doi:10.3390/healthcare14203554)
Supplement: Supplementary file 1 [file healthcare-14-03554-s001.zip › healthcare-4630775-supplementary.pdf]

Supplementary Materials

Stakeholder-Reported Requirements for Future Artificial Intelligence Decision Support in Periodontal Care: A Cross-Sectional Survey of Dentists, Physicians and Patients in Kazakhstan

Supplementary Material S1. CROSS checklist (Consensus-Based Checklist for Reporting of Survey Studies). The table reproduces all 40 items of the original checklist in its 19 sections with the original item wording (Sharma et al., J. Gen. Intern. Med. 2021, 36, 3179–3187, Supplement 3; reference [45] of the article). The right-hand column gives where the information is reported or, when an item does not apply or the information is not available, the reason. Section numbers refer to the article; S1–S6, Tables S1–S13 and Figure S1 to the Supplementary Materials.

Section/topic	Item	Item description (original CROSS wording)	Reported in (section, table, figure or supplement) or reason if not applicable
Title and abstract	1a	State the word "survey" along with a commonly used term in title or abstract to introduce the study's design.	Title ("A Cross-Sectional Survey"); Abstract, Methods ("Cross-sectional online surveys")
	1b	Provide an informative summary in the abstract, covering background, objectives, methods, findings/results, interpretation/discussion, and conclusions.	Abstract (structured: Background/Objectives, Methods, Results, Conclusions)
Introduction — Background	2	Provide a background about the rationale of study, what has been previously done, and why this survey is needed.	Sections 1.1–1.3
Introduction — Purpose/aim	3	Identify specific purposes, aims, goals, or objectives of the study.	Section 1.4 (objectives 1–5)
Methods — Study design	4	Specify the study design in the methods section with a commonly used term (e.g., cross-sectional or longitudinal).	Section 2.1 (cross-sectional, questionnaire-based study with three target populations)
Methods — Data collection methods	5a	Describe the questionnaire (e.g., number of sections, number of questions, number and names of instruments used).	Section 2.3 (three study-specific questionnaires; numbers of closed and open items for each); Supplementary Materials S2 (items and codes), S5 (online forms) and S6 (approved paper versions)
	5b	Describe all questionnaire instruments that were used in the survey to measure particular concepts. Report target population, reported validity and reliability information, scoring/classification procedure, and reference links (if any).	Section 2.3 (target groups, development, preliminary face/content validity only; no previously validated instrument used, so no reference links); scoring and classification: Section 2.6, Table S2-C and S3 codebooks; reliability of the patient symptom items: Section 3.5 and Table S3
	5c	Provide information on pretesting of the questionnaire, if performed (in the article or in an online supplement). Report the method of pretesting, number of times questionnaire was pre-tested, number and demographics of participants used for pretesting, and the level of similarity of demographics between pre-testing participants and sample population.	Section 2.3 (two review rounds by the working group; one pilot with 5 clinicians and 10 patients assessing comprehension and completion time). The demographics of the pilot participants and their similarity to the study sample are not reported
	5d	Questionnaire if possible, should be fully provided (in the article, or as appendices or as an online supplement).	Supplementary Material S6, Parts E–G (approved Russian paper questionnaires with consent forms); S5 (online forms as printed after collection, Kazakh/Russian); S2 (English translation with analysis codes); Table S6 (paper-to-online differences)
Methods — Sample characteristics	6a	Describe the study population (i.e., background, locations, eligibility criteria for participant inclusion	Section 2.2 (target populations, setting, original eligibility criteria—dentists >5

Section/topic	Item	Item description (original CROSS wording)	Reported in (section, table, figure or supplement) or reason if not applicable
		in survey, exclusion criteria).	years of practice; patients >20 years with K05.4—and the achieved-sample criteria actually applied); Figure 1; S6
	6b	Describe the sampling techniques used (e.g., single stage or multistage sampling, simple random sampling, stratified sampling, cluster sampling, convenience sampling). Specify the locations of sample participants whenever clustered sampling was applied.	Section 2.2 (non-probability convenience recruitment through the channels listed for each group). No cluster sampling was used
	6c	Provide information on sample size, along with details of sample size calculation.	Section 2.7 (no a priori calculation; precision of estimates and events per predictor reported)
	6d	Describe how representative the sample is of the study population (or target population if possible), particularly for population-based surveys.	Sections 2.2 and 2.7 (samples not representative; invitation denominators unknown); Section 4.6
Methods — Survey administration	7a	Provide information on modes of questionnaire administration, including the type and number of contacts, the location where the survey was conducted (e.g., outpatient room or by use of online tools, such as SurveyMonkey).	Sections 2.2 and 2.4 (self-administered Google Forms; links forwarded through professional chats, clinics, professional associations, treating clinicians and the inter-university platform). The number of contacts or reminders per respondent was not recorded
	7b	Provide information of survey's time frame, such as periods of recruitment, exposure, and follow-up days.	Section 2.4 (27 July–19 September 2026); Section 3.1 (distribution of submissions over time). Exposure and follow-up periods: not applicable (cross-sectional survey)
	7c	Provide information on the entry process: For non-web-based surveys, provide approaches to minimize human error in data entry. For web-based surveys, provide approaches to prevent "multiple participation" of participants.	Section 2.4 (no sign-in, IP or e-mail collection; single participation not technically prevented); Sections 2.5 and 3.1 (post-export screening for exact and near-duplicate records and submission timestamps; one exact duplicate removed)
Methods — Study preparation	8	Describe any preparation process before conducting the survey (e.g., interviewers' training process, advertising the survey).	Section 2.3 (questionnaire development, working-group review, Kazakh translation, pilot); Section 2.2 (dissemination channels). No interviewers were involved (self-administered)
Methods — Ethical considerations	9a	Provide information on ethical approval for the survey if obtained, including informed consent, institutional review board [IRB] approval, Helsinki declaration, and good clinical practice [GCP] declaration (as appropriate).	Section 2.9; Institutional Review Board Statement (Declaration of Helsinki; LBC Protocol No. 1/ID:312, 24 June 2026); Informed Consent Statement; S5 (electronic consent screens); S6 (approved consent forms)
	9b	Provide information about survey anonymity and confidentiality and describe what mechanisms were used to protect unauthorized access.	Sections 2.2, 2.4 and 2.9 (anonymous participation; no names, IP or e-mail addresses collected); S6 information sheets (coded data in protected folders, access restricted to the principal investigator and supervisor); Data Availability Statement
Methods — Statistical analysis	10a	Describe statistical methods and analytical approach. Report the statistical software that was used for data analysis.	Section 2.8 (methods; Python 3 with pandas, SciPy and statsmodels; SPSS syntax executed in GNU PSPP 2.0); Supplementary Material S4
	10b	Report any modification of variables used in the analysis, along with reference (if available).	Sections 2.5 and 2.6 (0/1 option indicators, ordinal scores, reverse coding, binary risk factors, maximum selected level, composite outcome with

Section/topic	Item	Item description (original CROSS wording)	Reported in (section, table, figure or supplement) or reason if not applicable
			reference [48]); S3 codebooks; re-derivation in S4
	10c	Report details about how missing data was handled. Include rate of missing items, missing data mechanism (i.e., missing completely at random [MCAR], missing at random [MAR] or missing not at random [MNAR]) and methods used to deal with missing data (e.g., multiple imputation).	Sections 2.4–2.6 ("cannot say" treated as missing, ≤7.5% per item, not imputed; unknown composite components assumed absent, with sensitivity analyses (c), (d), (f)); Table S2 (missing values per item). The missing-data mechanism was not formally assessed and no multiple imputation was performed
	10d	State how non-response error was addressed.	Not addressed: with forwarded open links the invited population and non-respondents are unknown, so non-response error could not be assessed (Sections 2.2 and 4.6)
	10e	For longitudinal surveys, state how loss to follow-up was addressed.	Not applicable (cross-sectional survey without follow-up)
	10f	Indicate whether any methods such as weighting of items or propensity scores have been used to adjust for non-representativeness of the sample.	No weighting or propensity-score methods were used (all methods are listed in Section 2.8); non-representativeness is stated in Sections 2.2, 2.7 and 4.6
	10g	Describe any sensitivity analysis conducted.	Section 2.8 (sensitivity analyses (a)–(h), combined (a)+(b) and check (b*), with the stage at which each was introduced)
Results — Respondent characteristics	11a	Report numbers of individuals at each stage of the study. Consider using a flow diagram, if possible.	Figure 1 (flow diagram); Sections 2.5 and 3.1
	11b	Provide reasons for non-participation at each stage, if possible.	Not possible: anonymous open links; non-participants were not identifiable (Section 2.2)
	11c	Report response rate, present the definition of response rate or the formula used to calculate response rate.	Not calculable: invitation denominators unknown (Sections 2.2 and 4.6)
	11d	Provide information to define how unique visitors are determined. Report number of unique visitors along with relevant proportions (e.g., view proportion, participation proportion, completion proportion).	Not determinable: no sign-in, IP addresses or form-view counts were collected (Section 2.4), so unique visitors and view/participation/completion proportions cannot be reported; duplicate and near-duplicate screening of submissions is reported in Sections 2.5 and 3.1
Results — Descriptive results	12	Provide characteristics of study participants, as well as information on potential confounders and assessed outcomes.	Section 3.1; Table 1; Section 3.5 and Table S2 (outcomes and behavioural and systemic factors); Table S13 (September response block)
Results — Main findings	13a	Give unadjusted estimates and, if applicable, confounder-adjusted estimates along with 95% confidence intervals and p-values.	Sections 3.2–3.5; Tables 2–6 and S1, S4, S5, S7, S9, S10 (unadjusted estimates with 95% CI and p values); Table 7 (adjusted odds ratios with 95% CI and p values)
	13b	For multivariable analysis, provide information on the model building process, model fit statistics, and model assumptions (as appropriate).	Section 2.8 (simultaneous entry of the predictors, indicator coding, events per predictor, VIF); Table 7 and its footnote (likelihood-ratio χ^2, Cox–Snell, Nagelkerke and McFadden R^2, apparent AUC, VIF)
	13c	Provide details about any sensitivity analysis performed. If there are considerable amount of	Section 3.6; Table 8; Table S8 (including check (b*)). No imputation

Section/topic	Item	Item description (original CROSS wording)	Reported in (section, table, figure or supplement) or reason if not applicable
		missing data, report sensitivity analyses comparing the results of complete cases with that of the imputed dataset (if possible).	was performed; complete-case analysis (f) is compared with the main analysis
Discussion — Limitations	14	Discuss the limitations of the study, considering sources of potential biases and imprecisions, such as non-representativeness of sample, study design, important uncontrolled confounders.	Section 4.6
Discussion — Interpretations	15	Give a cautious overall interpretation of results, based on potential biases and imprecisions and suggest areas for future research.	Sections 4.1–4.5 (interpretation); Section 4.7 (future research); Section 5
Discussion — Generalizability	16	Discuss the external validity of the results.	Section 4.6 (non-representative samples, unverified patient eligibility); Section 5
Other sections — Role of funding source	17	State whether any funding organization has had any roles in the survey's design, implementation, and analysis.	Funding statement: no external funding was received, so no funder had a role
Other sections — Conflict of interest	18	Declare any potential conflict of interest.	Conflicts of Interest statement
Other sections — Acknowledgements	19	Provide names of organizations/persons that are acknowledged along with their contribution to the research.	Acknowledgments (participating clinics, professional associations and respondents)

Supplementary Material S2. Questionnaire coding and English translation. The tables list the thematic items in abridged form, numeric analysis codes and Russian response labels used for coding. The post-collection form printouts in S5 provide complete later stems, options and displayed response types; they do not certify all collection-period settings. Original Russian participant-information sheets and an English translation are provided separately in S6. S6 also reproduces the three Russian paper questionnaires approved by the Local Bioethics Committee. The intended paper response types and paper-to-online differences in Table S6 follow these approved paper versions. Items marked † were intended as mutually exclusive choices or a single rating but were implemented as checkboxes and contain multiple selections. Demographic items preceded thematic questions. The open questions, clinical qualifiers and legal framing are preserved below because they affect interpretation.

Table S2-A. Dentist questionnaire (n = 33): closed items Q1–Q15 and open-ended questions Q16–Q20.

Item	Type	Stem and response options (Russian, as recorded in the export and codebook)	English translation
Q1	Multiple	Методы обследования для автоматизации: сбор жалоб и анамнеза; визуальная оценка десны; подвижность зубов; зондирование глубины карманов; пальпация лимфоузлов; перкуссия зубов	Examination procedures to automate first: history-taking and complaints; visual assessment of the gingiva; tooth mobility; probing of pocket depth; palpation of lymph nodes; percussion of teeth
Q2	Multiple	Рентгенологические признаки на ОПТГ: расширение периодонтальной щели; деструкция компактной пластинки; остеопороз межальвеолярных перегородок; глубина костных карманов; обнажение фуркаций; [все признаки в комплексе — указан в сравнении бумажной версии; отсутствует в поздней распечатке; наличие в период сбора не подтверждено]	Radiographic signs on panoramic radiographs: widening of the periodontal ligament space; destruction of the cortical plate; osteoporosis of the interalveolar septa; depth of bony pockets; furcation involvement; [all signs in combination — present in the approved paper version (S6); absent in the later printout; collection-period availability unverified]

Item	Type	Stem and response options (Russian, as recorded in the export and codebook)	English translation
Q3	Multiple	Коморбидные состояния в алгоритме ИИ: сахарный диабет; заболевания ЖКТ; сердечно-сосудистые; заболевания дыхательной системы; гормональные нарушения; остеопороз; иммунодефицитные состояния	Comorbid conditions to be considered by the AI algorithm: diabetes mellitus; gastrointestinal diseases; cardiovascular diseases; respiratory diseases; hormonal disorders; osteoporosis; immunodeficiency states
Q4	Multiple	Пародонтальные индексы, наиболее сложные для интерпретации без цифрового помощника: Грина–Вермиллиона; PMA; Рассела; Шиллера–Писарева; CPITN	Indices difficult to interpret without digital assistance: Greene–Vermillion; PMA; Russell; Schiller–Pisarev; CPITN
Q5	Multiple	Факторы риска для плана профилактики: стаж курения; качество гигиены; аномалии прикуса; качество пломб/протезов; стресс; экологические факторы	Risk factors for the prevention plan: smoking duration; quality of oral hygiene; malocclusion; quality of restorations/prostheses; stress; environmental factors
Q6	Multiple	Стадия, где ИИ наиболее востребован: скрининг здоровых; ранняя диагностика гингивита; дифдиагностика лёгкой/средней степени; оценка рисков при тяжёлых процессах; контроль ремиссии	Stage at which AI is most needed: screening of healthy individuals; early diagnosis of gingivitis; differential diagnosis of mild vs. moderate periodontitis; risk assessment in severe disease; monitoring of remission
Q7	Single	Какой уровень автономности ИИ-системы допустим при постановке диагноза «Пародонтит» согласно законодательству РК: низкая автономность; средняя автономность; СППР с обязательной верификацией; «второе мнение»; полная автономность запрещена, выбор всегда за врачом	Configuration considered permissible for diagnosing periodontitis under Kazakhstan legislation: low autonomy; medium autonomy; mandatory verification; second opinion; prohibition of full autonomy. Every option preserves clinician control. Legal permissibility and personal attitude are mixed; no autonomous option and no validated ordinal scale.
Q8	Multiple	Лабораторные данные для сопоставления: лейкоциты; цитокины (ИЛ-8, ИЛ-2); глюкоза > 6 ммоль/л; интерферон-альфа; [все показатели в динамике — указан в сравнении бумажной версии; отсутствует в поздней распечатке; наличие в период сбора не подтверждено]	Laboratory data to be matched with periodontal findings: leukocytes; cytokines (IL-8, IL-2); glucose > 6 mmol/L; interferon-alpha; [all indicators over time — present in the approved paper version (S6); absent in the later printout; collection-period availability unverified]
Q9	Multiple	Приоритетная функция ИИ: предиктивный анализ потери зубов; расчёт индексов по фото; дистанционный мониторинг; выбор антибактериальной терапии; геймификация/обучение гигиене	Priority AI function: predictive analysis of tooth loss; index calculation from photographs; remote monitoring; selection of antibacterial therapy; gamification/hygiene education
Q10	Multiple	Анатомические факторы риска: мелкое преддверие; аномальные уздечки; тяжи слизистой; скученность зубов; вторичные деформации	Anatomical risk factors: shallow vestibule; abnormal frenula; mucosal cords; tooth crowding; secondary deformities
Q11 †	Multiple (online); single (paper)	Частота переоценки рисков: раз в год при стандартном осмотре; два раза в год без соматической патологии; четыре раза в год при диабете или ССЗ; ежемесячно через приложение; только при обострении	Re-assessment: annually at routine examination; twice yearly without somatic disease; four times yearly with diabetes/CVD; monthly through an app; only at exacerbation. Options refer to different contexts; the form displayed the standard check-all-that-apply instruction

Item	Type	Stem and response options (Russian, as recorded in the export and codebook)	English translation
Q12	Multiple	Форма представления результата: текст с кодом МКБ-10; графическая карта пародонта; вероятностный прогноз, %; пошаговый алгоритм лечения; список доп. обследований	Preferred output format: text with ICD-10 code; graphical periodontal chart; probabilistic prognosis (%); step-by-step treatment algorithm; list of additional investigations
Q13	Single	Важнейший аспект прозрачности: видеть исходные данные; объяснение логики; эксплуатационные ограничения; маркировка синтетических результатов; разграничение ответственности	Most important aspect of transparency: access to the source data; explanation of the reasoning; operational limitations; labelling of synthetic results; delimitation of responsibility
Q14	Multiple	Критерии эффективности лечения: стабилизация высоты перегородок; уменьшение глубины карманов; отсутствие кровоточивости; снижение подвижности; восстановление жевательной эффективности	Criteria of treatment success: stabilisation of septal height; reduction in pocket depth; absence of bleeding; reduced mobility; restoration of masticatory efficiency
Q15	Multiple	Барьеры интеграции ИИ: загруженность, нет времени; сложный интерфейс, ИТ-навыки; недоверие к результатам; нет юридической базы/страхования ответственности; несоответствие оборудования; стоимость	Barriers to AI integration: workload, lack of time; complex interface, IT skills; distrust of automated results; no legal framework/liability insurance; equipment incompatibility; cost
Q16–Q20	Open-ended	Q16: дополнительные клинические данные для обучения модели перехода гингивита в пародонтит; Q17: взаимодействие стоматолога и интерниста через общую ИИ-платформу; Q18: подсказки/предупреждения при сложном хирургическом вмешательстве; Q19: ошибки заполнения медицинских карт ф. 043-у; Q20: распределение ответственности при расхождении диагноза ИИ и клинического заключения с развитием осложнений	Q16, additional clinical training data; Q17, dentist–internist interaction; Q18, useful intraoperative prompts/warnings; Q19, documentation errors in dental records (form 043-y); Q20, liability after discordant AI/clinician diagnoses and a complication. Retained substantive answers: 30, 27, 21, 25 and 29 (132 total), although all five items are required in the later printouts; the discrepancy in historical settings/preparation is unresolved. Q20 is tallied in S12 in its scenario-specific context

Table S2-B. Questionnaire for PHC physicians and medical specialists (n = 91): closed items Q4–Q13 (Q1–Q3: organisation, specialty, experience) and open-ended questions Q14–Q16.

Item	Type	Stem and response options (Russian, as recorded in the export and codebook)	English translation
Q4 †	Multiple (online); single ordinal (paper)	Критичность состояния пародонта для компенсации СД: крайне критично; очень важно; важно (индикатор ангиопатий); умеренно важно; маловажно	Importance of periodontal status for the compensation of diabetes: extremely critical; very important; important (indicator of angiopathy); moderately important; of little importance
Q5	Multiple	Анамнестические факторы тяжёлого пародонтита: стаж курения; длительность соматического заболевания; возраст > 40–50 лет; наследственность; стресс; низкая доступность стоматологической помощи	Anamnestic factors of severe periodontitis: smoking duration; duration of the systemic disease; age > 40–50 years; heredity; stress; low accessibility of dental care

Item	Type	Stem and response options (Russian, as recorded in the export and codebook)	English translation
Q6	Multiple	Коморбидности для автоматического предупреждения ИИ о риске потери зубов: сахарный диабет [нет в авторском сравнении бумажной версии]; заболевания ЖКТ; сердечно-сосудистые; болезни дыхательной системы; остеопороз; иммунодефициты; гормональные перестройки	Comorbidities for automatic AI alerts about the risk of tooth loss: diabetes mellitus [absent in the approved paper version]; gastrointestinal diseases; cardiovascular diseases; respiratory diseases; osteoporosis; immunodeficiency states; hormonal changes
Q7	Multiple	Изменения ОАК, требующие направления к пародонтологу: лейкоциты ↑/↓; тромбоцитопения; апластическая анемия/агранулоцитоз; незрелые формы клеток; СОЭ ↑ без очагов	Complete blood count findings warranting referral to a periodontist: leukocytosis/leukopenia; thrombocytopenia; aplastic anaemia/agranulocytosis; immature cell forms; raised ESR without a focus
Q8	Multiple	Жалобы, указывающие на гингивит: кровоточивость; галитоз; зуд и жжение; периодическая припухлость; дискомфорт при жевании	Complaints indicating gingivitis: gingival bleeding; halitosis; itching and burning; periodic swelling; discomfort on chewing
Q9	Multiple	Барьеры осмотра полости рта врачом ПМСП: нет времени; недостаток компетенций; нет простых инструментов (ИИ-сканер); зона ответственности стоматолога; низкая мотивация пациентов	Barriers to oral examination by a PHC physician: lack of time; insufficient competence; no simple tools (AI scanner); the dentist's area of responsibility; low patient motivation
Q10	Single	Данные о качестве жизни (DALY): число удалённых зубов; ограничения в выборе пищи; экстренные обращения; эстетика/психология; экономические затраты	Best indicator of the impact on quality of life (DALY): number of extracted teeth; restrictions in food choice; emergency visits; aesthetics/psychology; economic costs
Q11	Multiple	Стадия, на которой наиболее эффективно участие ПМСП: ранний скрининг гингивита; выявление факторов риска; контроль ремиссии; скрининг беременных; оценка перед плановыми операциями	Stage at which PHC involvement is most effective: early screening for gingivitis; identification of risk factors; monitoring of remission; screening of pregnant women; assessment before elective surgery
Q12	Multiple	Изменения пародонта при патологии ЖКТ: катаральные/язвенные процессы; стойкий налёт; изменение микробиоценоза; рецидивирующее течение; снижение резистентности слизистой	Periodontal changes in gastrointestinal disease: catarrhal/ulcerative processes; persistent plaque; altered microbiocenosis; recurrent course; reduced mucosal resistance
Q13	Multiple	Приоритетные группы ИИ-скрининга: пожилые ≥ 65 лет; пациенты с СД; работники вредных производств; курильщики > 5 лет; беременные	Priority groups for AI-assisted screening: adults ≥ 65 years; patients with diabetes; workers in hazardous industries; smokers > 5 years; pregnant women
Q14–Q16	Open-ended	Q14: соматические заболевания, сочетающиеся с тяжёлым поражением пародонта; Q15: данные о стоматологическом лечении, необходимые для коррекции врачебной тактики; Q16: взаимодействие стоматолога и врача-интерниста через общую ИИ-платформу	Q14, systemic diseases seen with severe periodontal destruction; Q15, dental-treatment data needed to adjust medical management; Q16, dentist–internist interaction through a shared AI platform. 273 free-text answers, 91 per item; illustrative quotations only

Table S2-C. Patient questionnaire (n = 455): items Q1–Q20 (preceded by sex, age and dispensary status). Scores in parentheses are the ordinal codes used in the analysis (Section 2.6).

Item	Type	Stem and response options (Russian, as recorded in the export and codebook)	English translation
Q1	Single (ordinal)	Кровоточивость дёсен: никогда; очень редко; при жёсткой пище; при каждой чистке; спонтанно; постоянно	Gingival bleeding: never (0); very rarely (1); when eating hard food (2); at every brushing (3); spontaneously (4); constantly (5). Composite threshold: score ≥ 2
Q2	Single (ordinal)	Неприятный запах изо рта: нет; только утром; периодически днём; часто; постоянно; затрудняюсь ответить	Halitosis: no (0); only in the morning (1); periodically during the day (2); often (3); constantly (4); cannot say (missing)
Q3 †	Multiple (online); single (paper)	Подвижность зубов: нет, стоят крепко; едва заметное движение 1–2 зубов; смещаются при надавливании; мешает жевать; сильно шатаются; зубы отсутствуют/полные протезы	Tooth mobility: no, teeth are firm (0); barely noticeable movement of 1–2 teeth (1); displaced on pressure (2); interferes with chewing (3); very loose (4); teeth absent/complete dentures (missing when the sole answer). Score = highest degree selected; composite component: any of options 2–5 (score ≥ 1)
Q4 †	Multiple (online); single (paper)	Визуальные изменения дёсен: бледно-розовая, плотная; покраснение, отёк; «щели» между зубами; синюшный оттенок; гнойнички/припухлости; ярко-красная полоса	Visual gingival changes: pale pink and firm (healthy); redness, swelling; "gaps" between teeth (recession); bluish tint; pustules/swellings; bright red band. Score = number of pathological signs (0–5); composite component: any of options 2–6
Q5	Single (ordinal)	Болевые ощущения: нет боли; при кислом/сладком; зуд и жжение; при смыкании/жевании; острая самопроизвольная; постоянная ноющая	Pain: no pain (0); with sour/sweet food (1); itching and burning (2); on closing/chewing (3); acute spontaneous (4); constant aching (5)
Q6	Single (ordinal)	Частота чистки зубов: > 2 раз в день; 2 раза в день; 1 раз в день; не каждый день; только перед визитом к стоматологу; не чищу	Toothbrushing frequency: > 2 times a day; 2 times a day; once a day; not every day; only before a dental visit; do not brush. Reverse-coded 0–5; "< 2 times/day" = options 3–6
Q7	Single	Курение: никогда не курил(а); бросил(а); редко (< 5 сигарет в день); стаж до 5 лет; стаж > 10 лет; нагревание табака/вейп	Smoking: never smoked; quit; rarely (< 5 cigarettes/day); smoking for up to 5 years; smoking for > 10 years; heated tobacco/vape. Status: never / former / current (options 3–6)
Q8 †	Multiple (online); single (paper)	Дополнительные средства гигиены: весь комплекс ежедневно; только флосс; только ополаскиватель; ёршики по рекомендации; очень редко; нет, излишне	Additional hygiene aids: the full complex daily; floss only; mouthwash only; interdental brushes as recommended; very rarely; no, unnecessary. Adjunctive-aid indicator = options 1–4; mechanical-aid indicator = options 1, 2, 4. The options imply different frequencies, so neither indicator measures a common frequency of regular use
Q9	Single (ordinal)	Уровень стресса: полное спокойствие; обычный; умеренный; высокий; хронический, панические атаки; глубокая депрессия	Stress level: complete calm (0); usual (1); moderate (2); high (3); chronic, panic attacks (4); deep depression (5). "High stress" = options 4–6
Q10	Single	Качество питания: сбалансированное; мягкая, углеводистая; фастфуд, газировка; недостаток витаминов; нерегулярное; жёсткие диеты	Diet: balanced; soft, carbohydrate-rich; fast food, soft drinks; vitamin deficiency; irregular; strict diets

Item	Type	Stem and response options (Russian, as recorded in the export and codebook)	English translation
Q11	Single (ordinal)	Давность первых признаков: < 1 месяца; 6 мес.–1 год; 2–5 лет; > 5–10 лет; с детства/юности; затрудняюсь ответить; не было симптомов	Time since first signs: < 1 month; 6 months–1 year; 2–5 years; > 5–10 years; since childhood/adolescence; cannot say (missing); never had symptoms (0)
Q12	Single (ordinal)	Посещение стоматолога: каждые 6 мес.; раз в год; раз в 2–3 года; только при сильной боли; > 5 лет назад; никогда (по поводу дёсен)	Dental attendance: every 6 months; once a year; once in 2–3 years; only with severe pain; > 5 years ago; never (for gum problems). Reverse-coded 0–5; "≥ once a year" = options 1–2
Q13	Single	Ранее проведённое лечение дёсен: только снятие камня; кюретаж; лоскутные операции; антибиотики; физиотерапия; не проводилось	Previous periodontal treatment: scaling only; curettage; flap surgery; antibiotics; physiotherapy; none
Q14	Single (ordinal)	Гноетечение: никогда; однократно после травмы; иногда при нажатии; регулярно при обострениях; постоянно; затрудняюсь ответить	Suppuration: never (0); once after trauma (1); sometimes on pressure (2); regularly at exacerbations (3); constantly (4); cannot say (missing). Composite threshold: score ≥ 2
Q15	Single	Курсы лечения дёсен в год: 0; 1; 2; ≥ 3; постоянное поддерживающее; народные средства	Periodontal treatment courses per year: 0; 1; 2; ≥ 3; continuous supportive care; folk remedies
Q16	Single	Семейный анамнез пародонтита: нет; у одного родителя; семейная проблема; потеря всех зубов к 40–50 годам; протезы у бабушек/дедушек; нет информации	Family history: none; periodontal disease/loose teeth in a parent; a family problem; relatives lost all teeth by age 40–50; grandparents wore dentures from an early age; no information (separate category). Positive = options 2–5; these are unverified, partly non-specific self-reports
Q17	Single	Сахарный диабет/глюкоза: норма; преддиабет; СД 2 типа (контролируемый); СД 1 типа; СД неконтролируемый; никогда не проверял(а)	Diabetes/blood glucose: normal; prediabetes; type 2 diabetes (controlled); type 1 diabetes; uncontrolled diabetes; never tested (kept as a separate category). Dysglycaemia = options 2–5
Q18	Single	Сердечно-сосудистые заболевания: нет; артериальная гипертензия; ИБС; инфаркт/инсульт; атеросклероз; врождённые пороки	Cardiovascular diseases: none; arterial hypertension; ischaemic heart disease; myocardial infarction/stroke; atherosclerosis; congenital defects. Cardiovascular disease = options 2–6
Q19	Single	Суставы/кости: нет; остеопороз; ревматоидный артрит; боли без диагноза; системные заболевания соединительной ткани; затрудняюсь ответить	Joints/bones: none; osteoporosis; rheumatoid arthritis; pain without a diagnosis; systemic connective-tissue diseases; cannot say
Q20	Single (ordinal)	Сухость во рту: нет; по утрам/после стресса; периодическая; постоянная; с жжением языка; после начала приёма лекарств	Xerostomia: no (0); in the morning/after stress (1); periodic (2); constant (3); with burning of the tongue (4); after starting medication (missing)

Supplementary Material S3. Codebooks and verification report, provided as the Excel workbook Supplementary_S3_Codebooks_and_verification.xlsx (version 6, 16 October 2026). Sheets: README; Codebook_dentists, Codebook_physicians and Codebook_patients (variables, item wording, codes, derivation rules and whether S4 re-derives each variable); Flags (the six patient contradiction flags—including fl_disp, defined as a mismatch between the dispensary-registration answer and the diagnosis entered in the following free-text item, which was not retained in the coded data—and the multiple-selection indicators); Composite_outcome (positive if bleeding score ≥ 2 or mobility score ≥ 1 or number of pathological gingival signs ≥ 1 or suppuration score ≥ 2; in the main analysis an unknown mobility or suppuration component is assumed absent, so that 19 outcome-indeterminate records are classified as negative); Analysis_samples (definition, n and events of the main and every sensitivity-analysis sample); and four sheets copied unchanged from the archived S4 run on the final cleaned coded datasets: S4_derivation_check (65 re-derived variables compared with the stored values; all identical), S4_verification (2573 numerical values printed in the article and Supplementary Materials compared with the recomputed values at the printed precision; all agree; the two Table 1 values on the city of physicians and patients are listed as not reproducible because city is not retained in the coded files), TableS4_Holm (unadjusted and Holm-adjusted p values of the 56 comparisons of Table S4) and Cross_check (44 key statistics from S4 and from SPSS syntax executed in GNU PSPP 2.0; 43 agree, and for one Fisher test PSPP uses a different definition of the two-sided p). The README sheet records the run date, the software versions and the SHA-256 checksums of the three input files. **Supplementary Material S4.** Python analysis code, provided as the file Supplementary_S4_analysis_code.py, together with the complete output of its archived run, Supplementary_S4_output.xlsx (Python 3.13 with pandas 3.0, NumPy 2.5, SciPy 1.18 and statsmodels 0.15). Input: the three final cleaned coded datasets (stomatologi_clean, specialisty_clean and pacienty_clean, as CSV exports of the SPSS files; one row per respondent; variable names and codes as in the S3 codebooks), available from the corresponding author on reasonable request. The script (1) re-derives 65 stored variables from the coded item responses—for patients the age group, the ordinal scores, the summed symptom index, the composite outcome, the binary risk factors, five of the six contradiction flags, the any-contradiction flag and the numbers of options selected in Q3, Q4 and Q8; for clinicians the experience groups, the numbers of options selected, the single-choice codes, the total numbers of options selected and the maximum selected criticality level—compares them with the stored values (output sheet "Derivation_check") and stops with an error if any value differs; it also derives the unknown-component, outcome-indeterminate, mechanical-cleaning, multiple-selection and five-rule contradiction indicators and all sensitivity-analysis samples. Stored variables that cannot be re-derived from the coded files are used as stored and documented in the header of the script and in S3: the patient contradiction flag fl_disp (the free-text diagnosis answers were not retained), the response-block indicator cluster_sep (submission timestamps are not part of the coded files) and the clinicians' specialty group (assignment of free-text "other" answers). (2) It reproduces Table 1 (except city), Tables 3 and 5–8, Tables S1–S5, S7–S11 and S13, the statistics quoted in Sections 2.4–3.6 and the export part of the response-type check of Table S6 (output sheet "TableS6_response_types"), with exact tests (Fisher; Fisher–Freeman–Halton by complete enumeration) and Holm adjustment within each family; the only random procedure, the percentile bootstrap of the effect size r (1000 resamples), uses a fixed seed (20260727). (3) It compares every number printed in these tables, the significance marks and the listed text statistics with the recomputed values at the printed precision (output sheet "Verification") and ends with an error if any value differs. In the archived run all 65 re-derived variables were identical and all 2573 compared values agreed; the "Derivation_check" and "Verification" sheets of that run are reproduced in S3 as "S4_derivation_check" and "S4_verification". These checks verify the analysis of the coded files; they do not verify the original cleaning decisions that produced those files.

Supplementary Material S5. Post-collection printouts of questionnaire forms, supplied as Supplementary_S5_Questionnaire_documentation.pdf. The three unchanged form printouts contain 16 dentist, 13 physician and 15 patient pages, preceded by a new documentation cover. They show information screens, four consent statements, demographic and thematic items and response types. The later printouts are predominantly bilingual in Kazakh and Russian, with some demographic options in Russian only. The authors' export comparison described predominantly Russian labels with some bilingual variants. In addition, all five dentist open questions are required in these printouts whereas the retained substantive-

answer counts are 30, 27, 21, 25 and 29 of 33. No dated configuration log reconciles these differences. S5 is therefore documentation of the post-collection form state, not an independently verified archive of every form setting used during collection. Item numbers restart by section; mapping is given in S2.

Supplementary Material S6. Participant-information materials and approved paper questionnaires, supplied as Supplementary_S6_Participant_information_v3.pdf (29 pages): a cover identifying the scope of the documents; Parts A–C, the three original Russian information sheets (patients, dentists, physicians/specialists); Part D, their English translation; and Parts E–G, the three Russian paper questionnaires approved by the Local Bioethics Committee, each preceded by its signature-based informed-consent form (blank template). The investigator's personal telephone number has been removed from the information sheets to protect personal data. The patient sheet and consent form specify age >20 years and K05.4 (periodontosis) and envisage periodontal-chart data. Those intended procedures are distinguished from the unverified diagnostic eligibility and absence of linked clinical data in the present survey analysis. In the electronic versions used for data collection (S5), the information, consent statements and questionnaire were combined in a single form, without signature.

Table S1. Response options of dentists whose frequency differed by professional experience at the nominal level (unadjusted $p < 0.05$; 7 of 69 comparisons), with Holm-adjusted p values.

**Item and response option**	**≤ 5 years (n = 15), n (%)**	**> 5 years (n = 18), n (%)**	**OR [95% CI]**	**p (Holm p)**	**φ**
Q10. Tooth crowding	7 (46.7)	17 (94.4)	19.43 [2.03; 185.73]	0.004 (0.312)	0.53
Q2. Furcation involvement	4 (26.7)	14 (77.8)	9.63 [1.95; 47.44]	0.005 (0.372)	0.51
Q10. Secondary deformities	5 (33.3)	14 (77.8)	7.00 [1.49; 32.82]	0.015 (1.000)	0.45
Q3. Immunodeficiency states	5 (33.3)	14 (77.8)	7.00 [1.49; 32.82]	0.015 (1.000)	0.45
Q8. Glucose > 6 mmol/L	7 (46.7)	16 (88.9)	9.14 [1.53; 54.54]	0.020 (1.000)	0.46
Q15. Distrust of automated results	11 (73.3)	6 (33.3)	0.18 [0.04; 0.82]	0.037 (1.000)	0.40
Q5. Quality of restorations/prostheses	8 (53.3)	16 (88.9)	7.00 [1.17; 41.76]	0.047 (1.000)	0.40

Fisher's exact test (two-sided); unadjusted p, with the Holm–Bonferroni-adjusted p across the 71 experience comparisons (69 response options and the two single-choice items Q7 and Q13) in parentheses. None of the differences is significant after adjustment (smallest adjusted $p = 0.312$), so the table lists nominal differences only and does not establish differences between experience groups. OR, odds of selecting the option with > 5 years of experience relative to ≤ 5 years (Woolf 95% CI; 0.5 added to each cell when a zero cell was present).

Table S2. Descriptive statistics of the ordinal patient items (higher score = less favourable state).

**Item**	**n**	**Missing**	**Me [Q1; Q3]**	**Min–max**
Gingival bleeding (0–5)	455	0	1 [0; 1]	0–5
Halitosis (0–4)	435	20	1 [0; 1]	0–4
Tooth mobility (0–4)	441	14	0 [0; 0]	0–4
Number of pathological gingival signs (0–5)	455	0	0 [0; 1]	0–4
Pain (0–5)	455	0	0 [0; 0]	0–5
Suppuration (0–4)	430	25	0 [0; 0]	0–4
Xerostomia (0–4)	450	5	0 [0; 1]	0–4
Toothbrushing, reverse-coded (0–5)	455	0	1 [1; 2]	0–5
Stress (0–5)	455	0	2 [1; 2]	0–5
Dental attendance, reverse-coded (0–5)	455	0	1 [1; 3]	0–5
Symptom duration (0–5)	421	34	0 [0; 2]	0–5

Missing values correspond to "cannot say" responses; for mobility, to the single option "teeth absent or replaced by complete dentures"; for xerostomia, to "after starting medication".

Table S3. Item characteristics of the candidate six-item symptom scale (patients with complete answers, n = 399).

Item	Corrected item–total correlation	Cronbach's α if item deleted
Gingival bleeding (0–5)	0.32	0.52
Halitosis (0–4)	0.33	0.49
Tooth mobility (0–4)	0.33	0.50
Number of pathological gingival signs (0–5)	0.38	0.49
Pain (0–5)	0.31	0.50
Suppuration (0–4)	0.19	0.55

Cronbach's α = 0.56 (95% CI 0.48–0.62, Feldt's method); standardised α = 0.57; α without the 2–4 September 2026 block (n = 203) = 0.62 (95% CI 0.54–0.70).

Table S4. Severity of individual symptoms according to behavioural and systemic factors: effect size r (p), Mann–Whitney U test, with Holm–Bonferroni adjustment across the 56 comparisons.

Factor	Bleeding	Halitosis	Mobility	Gingival signs	Pain	Suppuration	Xerostomia
Female sex	0.09* (0.046)	0.04 (0.414)	0.04 (0.458)	0.05 (0.304)	0.05 (0.302)	−0.06 (0.184)	0.06 (0.214)
Current smoking	0.00 (0.976)	0.16*† (< 0.001)	0.07 (0.118)	0.04 (0.391)	0.12* (0.008)	0.20*† (< 0.001)	0.13* (0.004)
Toothbrushing < 2 times/day	0.02 (0.711)	0.12* (0.016)	0.07 (0.155)	0.06 (0.195)	0.01 (0.824)	0.06 (0.196)	0.03 (0.490)
High stress level	0.10* (0.033)	0.15* (0.001)	0.11* (0.022)	0.14* (0.003)	0.10* (0.030)	0.01 (0.812)	0.09* (0.047)
Dysglycaemia	0.01 (0.816)	0.06 (0.191)	0.14* (0.005)	0.22*† (< 0.001)	0.11* (0.026)	0.04 (0.457)	0.24*† (< 0.001)
Cardiovascular disease	0.10* (0.035)	0.08 (0.090)	0.17*† (< 0.001)	0.19*† (< 0.001)	0.07 (0.160)	0.08 (0.115)	0.20*† (< 0.001)
Positive family history	0.16* (0.002)	0.22*† (< 0.001)	0.19*† (< 0.001)	0.15* (0.003)	0.19*† (< 0.001)	0.18*† (< 0.001)	0.30*† (< 0.001)
Dental visits ≥ once a year	0.01 (0.822)	−0.04 (0.359)	−0.05 (0.275)	−0.03 (0.511)	−0.06 (0.212)	−0.07 (0.121)	−0.02 (0.597)

r = Z/√N; r > 0 indicates a higher symptom score in the presence of the factor; * p < 0.05 (unadjusted); † significant after Holm–Bonferroni adjustment across the 56 comparisons (adjusted p < 0.05). The 12 Holm-significant associations are: current smoking with halitosis and suppuration (2); dysglycaemia with gingival signs and xerostomia (2); cardiovascular disease with mobility, gingival signs and xerostomia (3); positive family history with halitosis, mobility, pain, suppuration and xerostomia (5). None of the associations with high stress, toothbrushing, sex or dental attendance remained significant after adjustment (smallest adjusted p for high stress = 0.057, halitosis). Unadjusted and adjusted p values for all 56 comparisons are listed in the S3 workbook (sheet "TableS4_Holm").

Table S5. Severity of symptoms according to age group and smoking status (Kruskal–Wallis test), with Holm-adjusted p values.

Symptom	Age: H(3)	p (Holm p)	ε^2	Smoking: H(2)	p (Holm p)	ε^2
Bleeding	13.99	0.003 (0.032)	0.031	0.02	0.991 (0.991)	0.000
Halitosis	9.95	0.019 (0.152)	0.023	21.14	< 0.001 (< 0.001)	0.049
Mobility	5.56	0.135 (0.533)	0.013	2.66	0.265 (0.533)	0.006
Gingival signs	33.86	< 0.001 (< 0.001)	0.075	7.90	0.019 (0.152)	0.017
Pain	9.54	0.023 (0.152)	0.021	11.57	0.003 (0.032)	0.025
Suppuration	5.59	0.133 (0.533)	0.013	19.40	< 0.001 (< 0.001)	0.045

Symptom	Age: H(3)	p (Holm p)	ε²	Smoking: H(2)	p (Holm p)	ε²
Xerostomia	8.16	0.043 (0.214)	0.018	9.96	0.007 (0.062)	0.022

Age groups: 18–29, 30–44, 45–59, ≥ 60 years; smoking status: never, former, current. Unadjusted p, with the Holm–Bonferroni-adjusted p across the 14 comparisons of this table in parentheses: 10 comparisons were nominally significant and 5 after adjustment (age: bleeding and gingival signs; smoking: halitosis, pain and suppuration). $\varepsilon^2 = H/(n-1)$.

Table S6. Questionnaire implementation discrepancies and the scope of historical version verification. Paper-version details follow the approved paper questionnaires reproduced in S6; S5 is a later form state, not proof of all collection-period settings.

Questionnaire, item	Discrepancy	Respondents affected	Handling in the analysis	Consequence for interpretation
Dentists, Q11 (frequency of risk re-assessment)	No multi-select wording in the approved paper version (S6) or online stem; later printout is a checkbox item with the standard check-all-that-apply instruction	20 of 33 selected > 1 option	Analysed as multiple-choice (0/1 per option)	Frequencies have clinical/delivery qualifiers; multiple answers may concern different contexts. No single frequency can be inferred. Single-answer subset: 6 twice yearly, 5 four times yearly, 2 annually
Dentists, Q2 (radiographic signs)	Option "all signs in combination" is present in the approved paper version (S6), absent from later S5 printout and is coded only as an all-zero placeholder indicator (D2_6) carried over from the paper-version coding scheme	33 participants; collection-period availability unverified	Not analysed as a zero-frequency response; only coded individual options reported	Absence in the later printout does not establish when the option was omitted; response distributions cannot be reconstructed for an unrecorded option
Dentists, Q8 (laboratory data)	Option "all indicators over time" is present in the approved paper version (S6), absent from later S5 printout and is coded only as an all-zero placeholder indicator (D8_5) carried over from the paper-version coding scheme	33 participants; collection-period availability unverified	Not analysed as a zero-frequency response; only coded individual options reported	Absence in the later printout does not establish when the option was omitted; response distributions cannot be reconstructed for an unrecorded option

Questionnaire, item	Discrepancy	Respondents affected	Handling in the analysis	Consequence for interpretation
Physicians, Q4 (importance of periodontal status for diabetes control)	Single ordinal rating on paper; checkbox online with the instruction "you may select several options"	34 of 91 selected > 1 level; 16 of 42 selected "extremely critical" alone	Each level as 0/1; maximum selected level derived (S4_max) for group comparisons	Percentages overlap; the ordinal statistics assume that the highest selected level represents the respondent's view. Among the 57 single answers, "extremely critical" 16 (28.1%); comparisons by specialty and experience repeated on single answers (Section 3.3)
Physicians, Q6 (comorbidities for AI alerts)	Option "diabetes mellitus" present online, absent from the paper version	57 of 91 selected it	Coded answers retained; historical timing unverified	The paper version would not have allowed this comparison with dentists' Q3
Patients, Q3 (tooth mobility)	Single-choice on paper; checkbox online with the instruction "you may select several options"	10 of 455 selected > 1 option (6 combined "teeth are firm" with a degree of mobility)	Score = highest degree selected; flagged as contradiction when combined with "teeth are firm"	Contradictions resolved in favour of the reported sign; sensitivity analyses (b) and (g); single-answer distribution in Table S11
Patients, Q4 (visual gingival changes)	Single-choice on paper; checkbox online with the instruction "you may select several options"	39 of 455 selected > 1 option (23 combined "pale pink, firm" with a pathological sign)	Score = number of pathological signs; flagged as contradiction when combined with "pale pink, firm"	Contradictions resolved in favour of the reported sign; sensitivity analyses (b) and (g); single-answer distribution in Table S11
Patients, Q8 (additional hygiene aids)	Single-choice on paper; checkbox online with the instruction "you may select several options"	47 of 455 selected > 1 option (3 combined "unnecessary" with use of an aid)	Dichotomised: adjunctive aids (options 1–4) and, post hoc, mechanical aids (options 1, 2, 4)	Mouthwash-only users (n = 35) counted as users in the original indicator; sensitivity analyses (e) and (g); single-answer distribution in Table S11
Physicians, Q5–Q9 and Q11–Q13 (list items)	No multiple-choice instruction in the paper version; checkbox items with the instruction "you may select several options" online	51 to 65 of 91 selected > 1 option per item	Analysed as multiple-choice items (0/1 per option)	None beyond the general one: percentages within an item may sum to more than 100%

Questionnaire, item	Discrepancy	Respondents affected	Handling in the analysis	Consequence for interpretation
All forms, respondent characteristics	Added online: sex, age and city of work (dentists, physicians); city of residence (patients); consent confirmation with four statements (physicians); patient age asked as "How many full years are you?"	All respondents	Descriptive use; consent content documented, historical validation of all four checkboxes not independently verified	None
Dentists and physicians, position/specialty	Fixed options plus "other"; online, "other" answered as free text (e.g., orthopaedic dentist, orthodontist, endodontist; dermatologist)	Respondents choosing "other"	Free-text answers assigned to the specialty groups of Table 1 and Table S7	Grouping of free-text specialties by the authors
Physicians, open-ended questions	Two open-ended questions on paper; a third (interaction between dentist and internist through a common AI platform, as in the dentist questionnaire) added online	91 (all answered)	Quoted only as illustrations; no content analysis	None
All forms, language history	Later printouts are more extensively bilingual than the authors' description of exported labels	Language relied on not recorded	Describe both records; no retrospective language assignment	No verified chronology or language-equivalence validation
Dentists, Q16–Q20 required status	Later printouts mark all five required; retained substantive-answer counts are 30, 27, 21, 25, 29	33 dentists; 132 retained answers	Report per-item denominators; no invented account of missing answers	Historical settings and open-answer preparation do not reconcile from available documentation
Patient eligibility and linked records	Original information sheet states K05.4 and age >20 and envisages chart extraction; achieved analytic file contains no linked clinical record	455 retained, including 82 aged 18–20	No claim of confirmed diagnostic eligibility; age-restricted analysis (h)	The achieved sample is not a verified K05.4 cohort

Five items intended as single-choice or as a single rating contained multiple selections in the exported responses and were checkbox items in the online forms (Supplementary Material S5); the original submission stated six, which was an error. In total, 87 patients selected more than one option in at least one of Q3, Q4 and Q8 (37 of them also had an internal contradiction). The Russian wording of the options as recorded in the export and codebook is given in Tables S2-A to S2-C, and the complete bilingual forms in Supplementary Material S5.

Table S6 (continued). Response type of all closed thematic items in the online forms (Supplementary Material S5) and in the exported responses.

Questionnaire	Items intended as single-choice (approved paper versions, S6)	Items with multiple selections in the export (respondents with > 1 option)	Items with exactly one answer per respondent	Response type in the online form (S5)
Dentists (15 closed items; 12 multiple-choice)	Q7, Q11, Q13	Q11 (20 of 33)	Q7, Q13	Q11 checkbox with standard check-all-that-apply instruction, without extra stem wording; Q7/Q13 radio buttons; 12 other multi-select items checkboxes
Physicians (10 closed items; 8 multiple-choice)	Q4, Q10	Q4 (34 of 91)	Q10	Q4 checkbox with the instruction "you may select several options"; Q10 radio buttons; the 8 multiple-choice items checkboxes with the same instruction
Patients (20 closed items)	Q1–Q20	Q3 (10), Q4 (39), Q8 (47) of 455	Q1, Q2, Q5–Q7, Q9–Q20 (17 items)	Q3, Q4 and Q8 checkboxes with the instruction "you may select several options"; the other 17 items radio buttons

Of the 25 items intended as single-choice, 5 were checkbox items online and contained multiple selections in the export; the other 20 were radio-button items, and every respondent gave exactly one answer to them, so the online forms and the export agree for every item. The multiple-choice items of the clinician questionnaires all contained multiple selections. The export part of this check is reproduced by the analysis code (Supplementary Material S4, output sheet "TableS6_response_types"), in which the intended type of each item is taken from the approved paper versions (S6). This verifies the coded response patterns, not the historical required status, exact wording or language settings of the form. Lack of an explicit multi-select instruction in a paper list item alone does not establish that only one selection was conceptually intended.

Table S7. Principal findings of the physician survey by specialty group, n (%); Fisher–Freeman–Halton exact test across the four groups and Fisher's exact test for PHC physicians (general practitioners, internists and paediatricians; n = 62) versus medical specialists (n = 29), with Holm-adjusted p values in parentheses.

Item	GP/internists (n = 46)	Paediatricians (n = 16)	Endocrinologists (n = 7)	Other specialists (n = 22)	Exact p, 4 groups (Holm p)	PHC (n = 62) vs. specialists (n = 29)	p, Fisher (Holm p)
Q11: early screening for gingivitis	33 (71.7)	15 (93.8)	6 (85.7)	19 (86.4)	0.238 (1.000)	48 (77.4) vs. 25 (86.2)	0.406 (1.000)
Q11: identification of risk factors	20 (43.5)	12 (75.0)	4 (57.1)	12 (54.5)	0.180 (1.000)	32 (51.6) vs. 16 (55.2)	0.824 (1.000)
Q13: priority group — patients with diabetes	29 (63.0)	14 (87.5)	5 (71.4)	17 (77.3)	0.284 (1.000)	43 (69.4) vs. 22 (75.9)	0.623 (1.000)
Q13: priority group — adults ≥ 65 years	28 (60.9)	14 (87.5)	4 (57.1)	11 (50.0)	0.098 (1.000)	42 (67.7) vs. 15 (51.7)	0.167 (1.000)
Q13: priority group — hazardous industries	24 (52.2)	12 (75.0)	2 (28.6)	10 (45.5)	0.151 (1.000)	36 (58.1) vs. 12 (41.4)	0.178 (1.000)
Q13: priority group — smokers > 5 years	19 (41.3)	13 (81.2)	5 (71.4)	9 (40.9)	0.020 (0.261)	32 (51.6) vs. 14 (48.3)	0.824 (1.000)
Q9: barrier — insufficient competence	22 (47.8)	11 (68.8)	3 (42.9)	13 (59.1)	0.451 (1.000)	33 (53.2) vs. 16 (55.2)	1.000 (1.000)
Q9: barrier — low patient motivation	19 (41.3)	14 (87.5)	4 (57.1)	10 (45.5)	0.009 (0.142)	33 (53.2) vs. 14 (48.3)	0.822 (1.000)
Q9: barrier — lack of time	20 (43.5)	5 (31.2)	3 (42.9)	11 (50.0)	0.729 (1.000)	25 (40.3) vs. 14 (48.3)	0.503 (1.000)

Item	GP/internists (n = 46)	Paediatricians (n = 16)	Endocrinologists (n = 7)	Other specialists (n = 22)	Exact p, 4 groups (Holm p)	PHC (n = 62) vs. specialists (n = 29)	p, Fisher (Holm p)
Q9: barrier — no simple tool	14 (30.4)	10 (62.5)	5 (71.4)	9 (40.9)	0.049 (0.592)	24 (38.7) vs. 14 (48.3)	0.495 (1.000)
Q9: barrier — dentist's responsibility	16 (34.8)	8 (50.0)	1 (14.3)	7 (31.8)	0.412 (1.000)	24 (38.7) vs. 8 (27.6)	0.352 (1.000)
Q4: "extremely critical" for diabetes control	24 (52.2)	5 (31.2)	3 (42.9)	10 (45.5)	0.553 (1.000)	29 (46.8) vs. 13 (44.8)	1.000 (1.000)
Q6: alert condition — diabetes	28 (60.9)	14 (87.5)	4 (57.1)	11 (50.0)	0.098 (1.000)	42 (67.7) vs. 15 (51.7)	0.167 (1.000)
Q6: alert condition — gastrointestinal disease	26 (56.5)	15 (93.8)	3 (42.9)	17 (77.3)	0.010 (0.144)	41 (66.1) vs. 20 (69.0)	1.000 (1.000)
Q8: bleeding as a sign of gingivitis	33 (71.7)	15 (93.8)	5 (71.4)	17 (77.3)	0.313 (1.000)	48 (77.4) vs. 22 (75.9)	1.000 (1.000)

Four-group comparison: Fisher–Freeman–Halton exact test for 2 × 4 tables (each of the 15 tables had at least two cells with expected frequencies < 5, so the asymptotic Pearson χ^2 p values of the previous version were replaced by exact p values). Four comparisons were nominally significant (low patient motivation, gastrointestinal disease as an alert condition, smokers as a priority group and no simple tool); none was significant after Holm adjustment across the 15 comparisons (smallest adjusted p = 0.142), and none of the 15 PHC-versus-specialist comparisons was significant before or after adjustment. Absence of significant differences does not establish equivalence between groups. GP, general practitioner; PHC, primary health care.

Table S8. Sensitivity analyses of the multivariable logistic regression for self-reported periodontal signs: adjusted odds ratios [95% CI] for all predictors (B, SE and Wald statistics are reproducible with the code in Supplementary Material S4).

Predictor	Main model	(a) Without 2–4 Sep block	(b) Without contradictory records	(c) Without outcome-indeterminate records	(d) Outcome-indeterminate as positive	(e) Mechanical aids	(a) + (b)
Age (per 10 years)	1.11 [0.97; 1.28]	1.23 [0.99; 1.52]	1.10 [0.94; 1.28]	1.17 [1.01; 1.35]*	1.19 [1.03; 1.38]*	1.10 [0.95; 1.27]	1.24 [0.98; 1.55]
Female sex	1.34 [0.86; 2.09]	1.45 [0.75; 2.81]	1.29 [0.80; 2.07]	1.27 [0.81; 2.00]	1.20 [0.77; 1.87]	1.37 [0.88; 2.14]	1.26 [0.62; 2.54]
Former smoker (vs. never)	1.58 [0.80; 3.11]	1.58 [0.70; 3.55]	1.67 [0.82; 3.42]	1.49 [0.75; 2.99]	1.43 [0.72; 2.84]	1.67 [0.85; 3.31]	1.65 [0.71; 3.84]
Current smoker (vs. never)	0.85 [0.45; 1.60]	0.55 [0.25; 1.22]	0.94 [0.48; 1.85]	1.00 [0.51; 1.93]	1.08 [0.57; 2.07]	0.84 [0.44; 1.58]	0.53 [0.23; 1.23]
Toothbrushing < 2 times/day	1.04 [0.66; 1.63]	1.18 [0.64; 2.17]	0.86 [0.52; 1.41]	0.96 [0.61; 1.53]	0.91 [0.58; 1.43]	1.00 [0.63; 1.56]	1.06 [0.55; 2.04]
Hygiene aids (adjunctive; mechanical in (e))	0.75 [0.49; 1.14]	0.79 [0.45; 1.40]	0.72 [0.45; 1.14]	0.69 [0.45; 1.07]	0.69 [0.45; 1.05]	0.57 [0.37; 0.86]*	0.86 [0.46; 1.60]
High stress level	2.11 [1.14; 3.89]*	1.73 [0.78; 3.85]	2.33 [1.22; 4.48]*	1.83 [0.99; 3.41]	1.68 [0.91; 3.13]	2.08 [1.13; 3.85]*	1.93 [0.81; 4.60]
Dysglycaemia (vs. normal glucose)	2.02 [0.81; 5.04]	2.48 [0.75; 8.17]	2.22 [0.77; 6.38]	1.60 [0.63; 4.06]	1.44 [0.57; 3.64]	2.12 [0.85; 5.30]	2.57 [0.64; 10.28]
Glucose never tested (vs. normal)	2.18 [0.91; 5.25]	3.19 [0.88; 11.60]	2.12 [0.85; 5.26]	2.52 [0.98; 6.50]	2.60 [1.03; 6.58]*	2.28 [0.94; 5.50]	3.46 [0.95; 12.69]
Cardiovascular disease	1.72 [0.89; 3.34]	1.30 [0.59; 2.87]	1.59 [0.79; 3.18]	2.07 [1.02; 4.20]*	2.11 [1.05; 4.25]*	1.65 [0.85; 3.20]	1.05 [0.45; 2.45]
Positive family history (vs. none)	1.61 [1.02; 2.55]*	1.52 [0.82; 2.81]	1.56 [0.94; 2.60]	1.66 [1.04; 2.65]*	1.62 [1.02; 2.58]*	1.54 [0.96; 2.45]	1.65 [0.84; 3.23]
Family history unknown (vs. none)	1.18 [0.65; 2.16]	1.16 [0.55; 2.47]	1.17 [0.61; 2.24]	1.51 [0.79; 2.90]	1.76 [0.94; 3.29]	1.08 [0.59; 1.98]	1.18 [0.53; 2.62]
n (events)	455 (223)	251 (140)	393 (181)	436 (223)	455 (242)	455 (223)	216 (114)
Likelihood-ratio $\chi^2(12)$	44.11	25.29	39.01	48.19	51.00	49.48	22.55
Model p	< 0.001	0.014	< 0.001	< 0.001	< 0.001	< 0.001	0.032
Nagelkerke R^2	0.123	0.128	0.126	0.140	0.142	0.137	0.132
AUC (apparent)	0.665	0.674	0.664	0.680	0.683	0.675	0.676

** p < 0.05 (nominal, unadjusted; the analyses re-estimate one model under alternative assumptions and were not treated as a family of independent tests). Simultaneous entry; indicator coding with reference categories male sex, never smoked, normal glucose, no family history. (a) Exclusion of the 204 responses received on 2–4 September 2026; (b) exclusion of the 62 records with internal contradictions; (c) exclusion of the 19 outcome-indeterminate records (no observed positive component and at least one unknown component); (d) these 19 records reclassified as positive; (e) adjunctive-hygiene indicator replaced by the mechanical-interdental-cleaning indicator; (f) restriction to the 417 patients with all four components known; (g) exclusion of the 87 patients with multiple selections in Q3, Q4 or Q8; (h) restriction to patients aged > 20 years. Analysis (a) was specified after data inspection; (b)–(h) were added during peer review. AUC values are apparent (in-sample) without validation.*

Table S8 (continued). Additional sensitivity analyses (f)–(h): adjusted odds ratios [95% CI] for all predictors.

Predictor	(f) All four components known	(g) Without multiple selections (Q3, Q4, Q8)	(h) Age > 20 years
Age (per 10 years)	1.11 [0.95; 1.29]	1.09 [0.93; 1.27]	1.26 [1.06; 1.51]*
Female sex	1.29 [0.81; 2.04]	1.42 [0.87; 2.31]	1.32 [0.80; 2.18]
Former smoker (vs. never)	1.16 [0.56; 2.42]	1.72 [0.82; 3.59]	1.62 [0.80; 3.28]
Current smoker (vs. never)	0.99 [0.50; 1.94]	0.92 [0.46; 1.83]	0.75 [0.37; 1.52]
Toothbrushing < 2 times/day	0.93 [0.58; 1.50]	0.81 [0.49; 1.34]	1.11 [0.66; 1.84]
Hygiene aids (adjunctive; mechanical in (e))	0.70 [0.45; 1.10]	0.81 [0.51; 1.30]	0.74 [0.46; 1.20]
High stress level	1.97 [1.06; 3.66]*	2.33 [1.16; 4.70]*	2.87 [1.42; 5.82]*
Dysglycaemia (vs. normal glucose)	1.55 [0.60; 4.04]	1.39 [0.48; 3.97]	2.02 [0.78; 5.24]
Glucose never tested (vs. normal)	2.67 [1.04; 6.90]*	2.98 [1.11; 8.00]*	2.26 [0.81; 6.32]
Cardiovascular disease	2.19 [1.06; 4.51]*	1.74 [0.84; 3.59]	1.68 [0.82; 3.44]
Positive family history (vs. none)	1.68 [1.04; 2.71]*	1.69 [0.99; 2.88]	1.35 [0.80; 2.28]
Family history unknown (vs. none)	1.48 [0.76; 2.87]	1.45 [0.73; 2.86]	1.00 [0.52; 1.92]
n (events)	417 (204)	368 (163)	373 (182)
Likelihood-ratio $\chi^2(12)$	42.74	36.67	45.73
Model p	< 0.001	< 0.001	< 0.001
Nagelkerke R^2	0.130	0.127	0.154
AUC (apparent)	0.668	0.666	0.693

* $p < 0.05$ (nominal, unadjusted). Same predictors, coding and estimation as in the main model. AUC values are apparent (in-sample) without validation.

Table S8 (continued). Additional check (b*) using only the five reproducible contradiction rules, without fl_disp (n = 396; 183 events).

Predictor	aOR [95% CI]	Nominal p
Age (per 10 years)	1.11 [0.95; 1.29]	0.192
Female sex	1.32 [0.82; 2.12]	0.259
Former smoker (vs. never)	1.65 [0.80; 3.37]	0.172
Current smoker (vs. never)	0.89 [0.45; 1.74]	0.726
Toothbrushing < 2 times/day	0.85 [0.52; 1.40]	0.524
Hygiene aids	0.71 [0.45; 1.13]	0.153
High stress level	2.21 [1.16; 4.21]	0.016
Dysglycaemia (vs. normal glucose)	2.14 [0.75; 6.15]	0.156
Glucose never tested (vs. normal)	2.16 [0.87; 5.35]	0.097
Cardiovascular disease	1.74 [0.87; 3.46]	0.114
Positive family history (vs. none)	1.53 [0.92; 2.54]	0.101
Family history unknown (vs. none)	1.17 [0.61; 2.23]	0.644

The dispensary-status flag, which relied on a free-text diagnosis item not retained in the coded data, affected five records, three exclusively. In this check only the five rules reproducible from item codes are used. Apparent AUC = 0.665; no validation. This check supplements analyses (a)–(h) and is reproduced by S4 (Check_b_without_fl_disp).

Table S9. Spearman rank correlations between symptoms, age and behavioural factors in patients (pairwise n = 398–455), with Holm adjustment.

Variable	1	2	3	4	5	6	7	8	9	10	11	12
1. Age	1											
2. Bleeding	0.05	1										
3. Halitosis	−0.03	**0.30***	1									
4. Mobility	0.03	**0.22***	**0.18***	1								
5. Gingival signs	**0.23***	**0.24***	**0.22***	**0.34***	1							
6. Pain	0.06	**0.20***	**0.25***	**0.26***	**0.33***	1						
7. Suppuration	0.02	0.03	**0.16***	0.06	0.13*	**0.15***	1					
8. Xerostomia	0.09	**0.16***	**0.26***	**0.22***	**0.24***	**0.23***	**0.18***	1				
9. Brushing (reverse)	0.06	0.08	**0.18***	0.10*	0.11*	0.05	0.05	0.04	1			
10. Stress	−0.07	0.12*	**0.21***	0.13*	**0.19***	**0.16***	−0.00	**0.20***	0.11*	1		
11. Dental visits (reverse)	0.06	−0.05	0.06	0.04	0.03	0.03	0.04	0.02	**0.32***	0.10*	1	
12. Symptom duration	0.13*	**0.37***	**0.35***	**0.29***	**0.37***	**0.37***	**0.19***	**0.24***	−0.00	**0.18***	−0.06	1

* $p < 0.05$ (unadjusted); values in bold remained significant after Holm–Bonferroni adjustment across the 66 correlations of the matrix (adjusted $p < 0.05$). 41 correlations were nominally significant and 33 remained significant after adjustment; all were weak ($|\rho| \le 0.37$). Unadjusted and adjusted p values are reproduced by Supplementary Material S4 (output sheet "TableS9").

Table S10. Univariable associations with self-reported periodontal signs without the 2–4 September 2026 response block (n = 251; 140 with signs), compared with the full sample.

Factor	Factor present: n/N (%)	Factor absent: n/N (%)	OR [95% CI]	p (Holm p)	Full sample (n = 455): OR; Holm p
Female sex	107/185 (57.8)	33/66 (50.0)	1.37 [0.78; 2.41]	0.271 (0.813)	1.41; 0.350
Current smoking	20/43 (46.5)	120/208 (57.7)	0.64 [0.33; 1.23]	0.179 (0.716)	1.13; 1.000
Toothbrushing < 2 times/day	47/79 (59.5)	93/172 (54.1)	1.25 [0.73; 2.14]	0.422 (0.843)	1.17; 1.000
High stress level	28/40 (70.0)	112/211 (53.1)	2.06 [1.00; 4.27]	0.048 (0.289)	2.68; 0.003
Dysglycaemia	19/24 (79.2)	112/214 (52.3)	3.46 [1.25; 9.61]	0.012 (0.098)	3.52; 0.008
Cardiovascular disease	38/54 (70.4)	102/197 (51.8)	2.21 [1.16; 4.23]	0.015 (0.104)	2.83; 0.002
Positive family history	58/92 (63.0)	58/115 (50.4)	1.68 [0.96; 2.93]	0.069 (0.347)	2.17; 0.003
Dental visits ≥ once a year	72/131 (55.0)	68/120 (56.7)	0.93 [0.57; 1.54]	0.786 (0.843)	0.91; 1.000

Pearson's χ^2 test (all expected frequencies ≥ 5); OR with Woolf 95% CI; Holm adjustment across the eight comparisons. Patients who had never had their glucose measured or did not know their family history were excluded from the respective comparison.

Table S11. Items designed as single-choice but implemented with multiple selection online: distribution among all respondents and among respondents who selected a single option.

Item and option	All respondents, n (%)	Single-answer respondents, n (%)
Dentists Q11 (risk re-assessment): Once a year	8 (24.2)	2 (15.4)
Dentists Q11 (risk re-assessment): Twice a year	18 (54.5)	6 (46.2)
Dentists Q11 (risk re-assessment): Four times a year	24 (72.7)	5 (38.5)
Dentists Q11 (risk re-assessment): Monthly	14 (42.4)	0 (0.0)
Dentists Q11 (risk re-assessment): Only at exacerbation	5 (15.2)	0 (0.0)
Physicians Q4 (importance for diabetes control): Extremely critical	42 (46.2)	16 (28.1)
Physicians Q4 (importance for diabetes control): Very important	45 (49.5)	17 (29.8)
Physicians Q4 (importance for diabetes control): Important (indicator of angiopathy)	46 (50.5)	19 (33.3)
Physicians Q4 (importance for diabetes control): Moderately important	11 (12.1)	4 (7.0)

Item and option	All respondents, n (%)	Single-answer respondents, n (%)
Physicians Q4 (importance for diabetes control): Of little importance	5 (5.5)	1 (1.8)
Patients Q3 (tooth mobility): No, teeth are firm	354 (77.8)	347 (78.0)
Patients Q3 (tooth mobility): Barely noticeable movement of 1–2 teeth	83 (18.2)	75 (16.9)
Patients Q3 (tooth mobility): Displaced on pressure	5 (1.1)	3 (0.7)
Patients Q3 (tooth mobility): Interferes with chewing	3 (0.7)	3 (0.7)
Patients Q3 (tooth mobility): Very loose	3 (0.7)	3 (0.7)
Patients Q3 (tooth mobility): Teeth absent/complete dentures	18 (4.0)	14 (3.1)
Patients Q4 (gingival appearance): Pale pink and firm	340 (74.7)	317 (76.2)
Patients Q4 (gingival appearance): Redness, swelling	47 (10.3)	31 (7.5)
Patients Q4 (gingival appearance): "Gaps" between teeth	85 (18.7)	51 (12.3)
Patients Q4 (gingival appearance): Bluish tint	10 (2.2)	5 (1.2)
Patients Q4 (gingival appearance): Pustules/swellings	7 (1.5)	5 (1.2)
Patients Q4 (gingival appearance): Bright red band	11 (2.4)	7 (1.7)
Patients Q8 (additional hygiene aids): Full complex daily	112 (24.6)	104 (25.5)
Patients Q8 (additional hygiene aids): Floss only	121 (26.6)	90 (22.1)
Patients Q8 (additional hygiene aids): Mouthwash only	58 (12.7)	26 (6.4)
Patients Q8 (additional hygiene aids): Interdental brushes as recommended	22 (4.8)	11 (2.7)
Patients Q8 (additional hygiene aids): Very rarely	135 (29.7)	122 (29.9)
Patients Q8 (additional hygiene aids): No, unnecessary	58 (12.7)	55 (13.5)

Denominators: dentists 33 (single answers 13); physicians 91 (57); patients 455 (Q3, 445; Q4, 416; Q8, 408). Percentages among all respondents may sum to more than 100% within an item.

Table S12. Dentists' open-ended answers on responsibility following discordant AI and clinician diagnoses and a patient complication (Q20): descriptive tally (29 of 33 dentists answered).

Category	n	Answers (verbatim Russian with English translation)
Responsibility with the clinician	20	«Ответственность на враче» (responsibility lies with the doctor); «Ответственность целиком на враче» (responsibility lies entirely with the doctor); «Окончательное решение всегда принимает врач, а ИИ только помогает. Если ошибка произошла из-за сбоя ИИ, ответственность должен нести и разработчик системы» (the final decision is always made by the doctor, and AI only helps; if an error results from an AI failure, the developer should also bear responsibility)
Shared or split responsibility	3	«50/50» (2 answers); «За врачом лечение, за ИИ прогноз» (treatment is the doctor's, prognosis is the AI's)
Other or unclassifiable	6	«Консилиум» (a council of doctors); «ИИ, только для прогноза» (AI only for prognosis); «Все индивидуально» (it depends on the case); «Исключить» (exclude); «Затрудняюсь ответить» (cannot say); «Да» (yes)

Answers were assigned to three predefined categories by reading the verbatim text; this is a descriptive tally, not a formal qualitative analysis, and no inter-coder agreement was assessed. One answer in the first category also assigned responsibility to the developer for system failures. The answers concern this hypothetical discordance-and-complication scenario and are not a general measure of preferred AI autonomy.

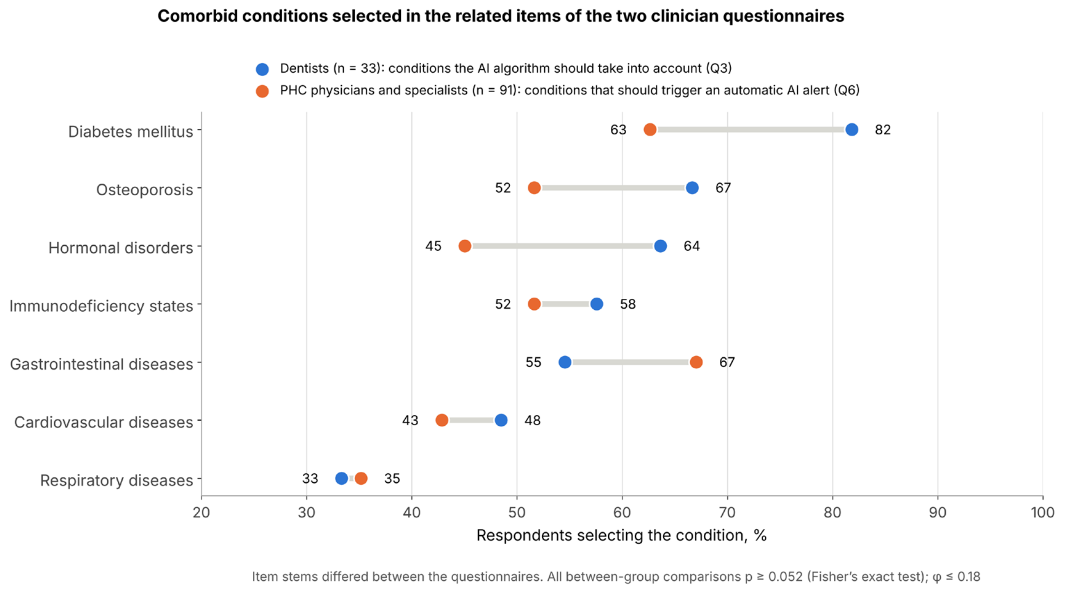

Figure S1. Comorbid conditions to be taken into account by an AI algorithm (dentists) or to trigger an automatic AI alert (physicians): comparison of dentists (n = 33) and PHC physicians/specialists (n = 91). The item stems differed between the two questionnaires (Section 3.4). Points show the percentage of respondents selecting each condition; p values from Fisher's exact test (Table 5 of the main text).

Table S13. Characteristics of the 2–4 September 2026 response block and the remaining patient sample. Values are n/N (%) unless indicated.

Characteristic	Block (n=204)	Remainder (n=251)	$\chi^2(1)$	Nominal p
Male sex	82/204 (40.2)	66/251 (26.3)	9.91	0.002
Any self-reported periodontal sign	83/204 (40.7)	140/251 (55.8)	10.26	0.001
Positive family history (known only)	37/188 (19.7)	92/207 (44.4)	27.47	<0.001
Current smoking	15/204 (7.4)	43/251 (17.1)	9.67	0.002
Dysglycaemia (known only)	8/192 (4.2)	24/238 (10.1)	5.40	0.020
Cardiovascular disease	10/204 (4.9)	54/251 (21.5)	25.69	<0.001
Toothbrushing ≥2/day	146/204 (71.6)	172/251 (68.5)	0.50	0.482
Adjunctive hygiene aid use	141/204 (69.1)	136/251 (54.2)	10.54	0.001
Balanced diet	86/204 (42.2)	123/251 (49.0)	2.12	0.145
Dental visits ≥annually	113/204 (55.4)	131/251 (52.2)	0.46	0.496
On dispensary register	6/204 (2.9)	36/251 (14.3)	17.46	<0.001
At least one contradiction flag	27/204 (13.2)	35/251 (13.9)	0.05	0.826

Age: median 22 [20; 42] vs. 44 [36; 57] years; U for the older remainder = 41,751, equivalent to the smaller U = 9453 shown in the supplied SPSS output; p <0.001, r = 0.55 [0.48; 0.61] (percentile bootstrap, S4). Age 20–22 or exactly 42: 190/204 vs. 14/251; age 18–20 (protocol-ineligible): 76/204 vs. 6/251. χ^2 tests are Pearson without continuity correction. Comparisons describe the observed block; nominal p values are exploratory and do not establish the mechanism of clustering or unique participation. Unknown family history and never-tested glucose are omitted from their respective denominators.
